# Computational estimation of tricarboxylic acid cycle fluxes using noisy NMR data from cardiac biopsies

**DOI:** 10.1186/1752-0509-7-82

**Published:** 2013-08-21

**Authors:** Hannes Hettling, David J C Alders, Jaap Heringa, Thomas W Binsl, A B Johan Groeneveld, Johannes H G M van Beek

**Affiliations:** 1 Centre for Integrative Bioinformatics (IBIVU), Vrije Universiteit Amsterdam, de Boelelaan 1081A, 1081 HV Amsterdam, The Netherlands; 2 Department of Anesthesiology, Leiden University Medical Center, PO Box 9600, 2300 RC Leiden, The Netherlands; 3 Department of Intensive Care, Erasmus Medical Center, PO Box 2040, 3000 CA Rotterdam, The Netherlands; 4 Department of Clinical Genetics, Section Functional Genomics, VU University Medical Center, Van der Boechorststraat 7, 1081 BT Amsterdam, The Netherlands; 5 Netherlands Consortium for Systems Biology (NCSB), Amsterdam, The Netherlands; 6 Netherlands Bioinformatics Centre (NBIC), Nijmegen, The Netherlands

**Keywords:** Cardiac physiology, Metabolic modeling, Metabolomics, Sensitivity analysis, ^13^C metabolic flux analysis

## Abstract

**Background:**

The aerobic energy metabolism of cardiac muscle cells is of major importance for the contractile function of the heart. Because energy metabolism is very heterogeneously distributed in heart tissue, especially during coronary disease, a method to quantify metabolic fluxes in small tissue samples is desirable. Taking tissue biopsies after infusion of substrates labeled with stable carbon isotopes makes this possible in animal experiments. However, the appreciable noise level in NMR spectra of extracted tissue samples makes computational estimation of metabolic fluxes challenging and a good method to define confidence regions was not yet available.

**Results:**

Here we present a computational analysis method for nuclear magnetic resonance (NMR) measurements of tricarboxylic acid (TCA) cycle metabolites. The method was validated using measurements on extracts of single tissue biopsies taken from porcine heart *in vivo*. Isotopic enrichment of glutamate was measured by NMR spectroscopy in tissue samples taken at a single time point after the timed infusion of ^13^C labeled substrates for the TCA cycle. The NMR intensities for glutamate were analyzed with a computational model describing carbon transitions in the TCA cycle and carbon exchange with amino acids. The model dynamics depended on five flux parameters, which were optimized to fit the NMR measurements. To determine confidence regions for the estimated fluxes, we used the Metropolis-Hastings algorithm for Markov chain Monte Carlo (MCMC) sampling to generate extensive ensembles of feasible flux combinations that describe the data within measurement precision limits. To validate our method, we compared myocardial oxygen consumption calculated from the TCA cycle flux with *in vivo* blood gas measurements for 38 hearts under several experimental conditions, e.g. during coronary artery narrowing.

**Conclusions:**

Despite the appreciable NMR noise level, the oxygen consumption in the tissue samples, estimated from the NMR spectra, correlates with blood-gas oxygen uptake measurements for the whole heart. The MCMC method provides confidence regions for the estimated metabolic fluxes in single cardiac biopsies, taking the quantified measurement noise level and the nonlinear dependencies between parameters fully into account.

## Background

Metabolic fluxes in animal tissues can be identified by measuring the incorporation of stable isotopes in intracellular metabolite pools. To quantify metabolic fluxes, isotope label incorporation is usually measured at several time points [1], among others in heart tissue [2]–[4]. Heterogeneity of metabolism inside the heart often confounds time series of small tissue samples, therefore a single time point protocol to quantify metabolic fluxes has been developed [5,6]. Such single time point measurements in individual samples allow to define spatial profiles of metabolic fluxes in heterogeneous organs [7].

The incorporation of stable isotopes (e.g. ^13^C) in metabolic intermediates can be detected by nuclear magnetic resonance (NMR) spectroscopy or mass spectrometry (MS). The data is then analyzed with computational methods that require (i) detailed mathematical models of carbon transitions between the metabolites in the system and (ii) sophisticated optimization procedures for estimating the flux parameters. In the past, we have developed a bioinformatics method to estimate metabolic fluxes in aerobic metabolism from very noisy NMR measurements resulting from the Labelling with Isotope for a Pre-Steady-State Snapshot (LIPSSS) protocol [8]. For LIPSSS, isotope labeled substrate for a metabolic pathway is infused for a short, definite period of time, and the metabolism is stopped before a steady state of label incorporation is reached. Finally, pathway metabolites are extracted and measured. Although the original computational analysis method [8] explores parameter space extensively to avoid local minima, only a rough estimate of parameter confidence regions was obtained by assuming local linearity. Here we introduce a Markov chain Monte Carlo (MCMC) parameter estimation strategy which allows a full description of the confidence regions of the estimated metabolic fluxes, including correlations and nonlinear dependencies between parameter estimates.

Brown et al. [9] and Gutenkunst et al. [10] sampled ensembles of parameter sets for systems biology models with MCMC. Correlations between model parameters were taken into account and confidence bounds for parameters and model predictions were defined [9,10]. Monte Carlo methods have previously been applied to metabolic flux analysis (MFA) in order to handle inaccuracies in data and model [11]. Sensitivity analysis by Monte Carlo sampling is also implemented in a ^13^C MFA analysis software package [12]. In ^13^C MFA, MCMC sampling has been used for uncertainty analysis [13,14], for flux estimation with noisy data [15], and for *in silico* experimental design to determine optimal substrate labeling protocols [16]. Antoniewicz et al. proposed a different approach of determining confidence bounds on fluxes by calculating the agreement between model and experiment data as a function of the flux of interest [17].

We developed and applied an MCMC procedure to estimate the TCA cycle flux, carbon substrate uptake, and oxygen consumption from NMR spectra of ^13^C enriched glutamate sampled at a single time point. For the computational analysis, we expanded the R-package FluxEs [8]. This analysis was applied to cardiac tissue biopsies flash-frozen 5.5 minutes following ^13^C acetate infusion in porcine hearts *in vivo*. The method was validated experimentally for a range of cardiac stress conditions. Our first goal was therefore to determine the uncertainty in the estimation of metabolic flux parameters based on the quantified uncertainty in the NMR measurements and in the prior knowledge. The second goal was to validate the computational estimations in experiments *in vivo*.

## Methods

### Ethical statement

The study was approved by the Advisory Board for the Use of Experimental Animals of the Vrije Universiteit Amsterdam. The procedure is in accordance with the American Physiological Society “Guiding Principles in the Care and Use of Animals,” which state that muscle relaxants may be used in conjunction with drugs known to produce adequate anesthesia.

### Experimental strategy

In this study the metabolic flux in the TCA cycle was measured in tissue biopsies taken from cardiac tissue via the LIPSSS experimental protocol which consists of a brief, timed infusion of ^13^C labeled acetate in the left anterior descending (LAD) coronary artery of anesthetized pigs [18]. We began with unlabeled acetate which was infused for 30 minutes, in order to establish a stationary metabolic state, followed by [2-^13^C] acetate for 4 minutes and [1,2-^13^C] acetate for 1.5 minutes. After exactly 5.5 minutes of ^13^C enriched acetate infusion, metabolism was arrested by freeze-clamping part of the left ventricular wall of the heart before the isotopic steady state was reached. Biopsies from different regions of this part of the left ventricular wall were cut from the tissue slab after freeze-drying, and divided into approximately nine samples per heart with around 0.1 g dry mass per sample. After extraction with perchloric acid, the ^13^C NMR multiplets of glutamate were measured. ^13^C-NMR spectra were obtained at 100.62 MHz and analyzed with the MRUI/AMARES software package (more information about tissue preparation, NMR measurement and the package can be found in reference [18]).

Up to nine separate multiplet intensities were detected for glutamate. For independent testing of the LIPSSS method and the associated parameter estimation procedures, “gold standard” myocardial oxygen uptake was calculated from blood flow, hemoglobin content and blood-gas measurements taken before and during acetate infusion [18]. Note that these classic oxygen uptake measurements are entirely independent of the LIPSSS method. We analyzed data from LIPSSS samples taken from N = 38 porcine hearts divided into 6 different experimental groups: (i) basal state of the heart (control group, n = 7), two groups with constriction (see below for method) of the coronary vessels to reduce blood flow ((ii) mild stenosis group, n = 7 and (iii) a moderate stenosis group, n = 6), (iv) peripheral venous infusion of dobutamine to induce cardiac stress (dobutamine group, n = 6) or (v) infusion of adenosine for cardiovascular dilatation (adenosine group, n = 4) and (vi) finally, a combination of stenosis and adenosine administration (stenosis + adenosine group, n = 8). In the mild and moderate stenosis groups, LAD blood pressure was adjusted with an occluder to amount to about 70 and 35 mmHg downstream of the occluder, respectively. In the adenosine and stenosis + adenosine groups, adenosine was infused into the LAD at a rate of 100 μg/kg/min. In the stenosis + adenosine group coronary blood pressure was reduced to about 45 mmHg. In the dobutamine group, dobutamine was infused at a rate of 10 μg/kg/min. Note that the dobutamine group initially contained 8 hearts from which two were excluded from further analysis, due to a low mean arterial blood pressure and insufficient NMR signal for parameter estimation (see [8]), respectively.

### Anesthesia and animal experimental procedures

In all groups, sedation was performed with ketamine 15 mg/kg and midazolam 1 mg/kg intramuscularly, and anesthesia was maintained by continuous infusion of sufentanil (4 μg/kg/hr), midazolam (0.5 mg/kg/hr), and pancuronium (0.2 mg/kg/hr). The trachea was intubated and the lungs were ventilated with a mixture of 60% oxygen/40% air. Fluid-filled catheters were introduced and hemodynamic parameters collected as previously described (see [18]). A continuous infusion of lidocaine was started to help prevent cardiac arrhythmias (9 mg/kg/hr, with an initial bolus injection of 50 mg). Five cm H_2_O of positive end-expiratory pressure (PEEP) was applied before opening the thorax. The thorax was opened via a midsternal incision and the heart exposed by opening the pericardium. The left hemiazygos vein was tied off to prevent mixing of noncoronary venous blood with coronary venous blood. The LAD was dissected free over a distance of about 2 cm and was catheterized with a 24G catheter. In the stenosis and adenosine + stenosis groups, a custom-made adjustable aluminium occluder was placed around the artery, and LAD pressure was measured.

After finishing instrumentation the animal was allowed to stabilize for at least 15 minutes, the first batch of microspheres (labeled with ^141^Ce or ^103^Ru, in random order) was injected into the left atrium for baseline blood flow measurements. The intervention was performed and 30 minutes later a second batch of microspheres was injected for final blood flow measurements. Throughout the procedure hemodynamic data were recorded continuously.

Experimental procedures have been described more extensively previously [7,18].

### Computational model

**Figure 1:**
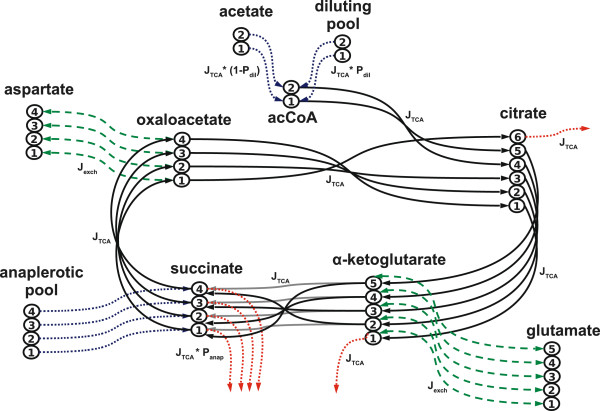
**Computational model of carbon transitions in the TCA cycle.** The numbered circles connected in a string represent single carbon atoms of the corresponding metabolite. Fluxes between carbon atoms of the metabolite pools are indicated by arrows. Blue and red dotted arrows stand for carbon atoms entering and leaving the system, respectively. Green dashed arrows indicate bidirectional exchange fluxes of carbon atoms with amino acids. The parameters determining the conversion rate are shown next to the arrows. Note that there are two possible transitions between α-ketoglutarate and succinate, indicated by arrows of different grey shade. The figure was adapted from Binsl et al. [8].

The NMR measured enrichment of glutamate with isotopes was analyzed with a computational model. The model of carbon transitions in the TCA cycle used in this study was described previously in detail [5,6,8] and is therefore only described here in brief. The model contains ten metabolite pools, consisting of metabolites which contain 2–6 carbon atoms, and 50 transitions of carbon atoms between the metabolites (Figure 1). Isotopically labeled substrate enters the system via the acetate pool. Acetate is then converted into acetyl coenzyme A (acetyl-CoA), which then enters the TCA cycle. Since acetyl-CoA can also be formed from endogenous unlabeled substrates such as glucose, glycogen, or fatty acids, a diluting pool was introduced to account for dilution of the labeled acetate. The intermediates of the TCA cycle are represented by the 6-carbon metabolite pool labeled as citrate (which also comprises cis-aconitate and isocitrate), α-ketoglutarate, succinate (including succinyl-CoA) and oxaloacetate (representing a 4-carbon metabolite pool which also comprises malate and fumarate). Glutamate and aspartate are amino acids synthesized by transamination from α-ketoglutarate and oxaloacetate, respectively. The replenishment of TCA cycle intermediates was modeled by an anaplerotic influx connected to succinate. Malloy et al. have given detailed descriptions of the equations for anaplerosis [3,19]. The metabolite concentrations were given as fixed parameters in the calculations: the glutamate pool size was measured by biochemical assay in each sample [18], because sensitivity analysis showed that results are sensitive to its value. However, the same sensitivity analysis showed that the metabolite pool concentrations of citrate, α-ketoglutarate, oxaloacetate and aspartate had little effect on the results and these concentrations were taken from previous studies [6,18]. All metabolite concentration parameters were therefore fixed and all flux parameters estimated during the Markov chain Monte Carlo procedure (see below). More information about the model and a listing of all model equations can be found in Additional file 1.

The dynamic behavior of the model is affected by five system parameters (Figure 1). The flux parameters J_TCA_ and J_exch_, were expressed in μmol/(min*g dry weight [dw]) and represent reaction fluxes through the TCA cycle and exchange reactions with amino acids, respectively. The dynamics of incorporation of ^13^C label from acetate into the acetyl-CoA pool depends on transport in the blood vessels, permeation of the cell membrane, the flux of the conversion of acetate into acetyl-CoA, the flux of acetyl-CoA into the TCA cycle and the acetate and acetyl-CoA pool sizes. Fortunately, the time course of incorporation of ^13^C label into the acetate pool is almost mono-exponential [20] and can be represented by a single time constant which we term T_trans_. We incorporated this efficient way to represent acetyl-CoA dynamics into our model [6]. The two parameters P_dil_ and P_anap_ account for the degree of dilution of labeled acetate and the rate of anaplerosis relative to TCA cycle flux, respectively. Both are flux parameters which are expressed as fractions of J_TCA_. J_TCA_ and P_dil_ describe energy and substrate turnover which are our targets to measure and are therefore labeled “primary parameters”. On the other hand, J_exch_, T_trans_ and P_anap_ are constrained during parameter estimation by Bayesian priors (see below) and because they are not our primary target parameters they are termed “auxiliary parameters” which are allowed to vary to determine the uncertainty which they cause in the primary parameters, The LIPSSS estimate for myocardial oxygen consumption is calculated from the primary parameters, (see Eq. 5 below). Note that primary and auxiliary parameters are estimated together in the same procedure.

### Matching model simulations to NMR measurements

The computational model described above accounts for all possible carbon isotope labeling states (isotopomers) of each of the metabolites. The system is described by 132 ordinary differential equations (ODEs) to calculate the rate of change of each isotopomer over time. For instance, the metabolite glutamate, which contains 5 carbons, is represented by 2^5^ = 32 ODEs. The isotopomer composition is expressed as fractions of the metabolite concentration of the corresponding pool. Thus, at each time point, the sum of all isotopomer fractions in a pool is 1. All ODEs are then integrated over time to yield the simulated isotopomer fractions. For comparison with the ^13^C NMR measurements (m_exp_), simulated NMR multiplet intensities (m_sim_) were calculated from the simulated isotopomer fractions for the time point at which the sample was taken in the experiment [8]. To this end all isotopomers contributing to a particular NMR intensity were added. The simulated multiplet intensities are dependent on the values of the five model parameters. To quantify the agreement between model simulation and experimental data we define a least-squares cost function C as a function of the parameter vector θ, in which the squared residuals for all multiplets are weighted by their standard deviations and summed. Additionally, we include Bayesian prior terms in the cost function which reflect prior knowledge on auxiliary parameter values (see below):

(1) $$C\left(\theta \right)=\frac{1}{2}\underset{i\in \mathrm{multiplets}}{\sum }{\left(\frac{m_{i,\mathrm{sim}}-m_{i,\exp}}{\sigma_{i,\exp}}\right)}^{2}+\underset{j\in \theta }{\sum }\mathrm{prior}\left(\theta_{j}\right)$$

The σ_i,exp_ represents the measurement error of the NMR intensity. This cost function is used for the optimization procedures. It is also used as the argument of the normal probability distribution used for the MCMC procedure (see below). The cost function integrates data measured directly in the experiment with literature information incorporated in the priors on parameter values.

### Priors on parameter values

The main objective of this study was to estimate J_TCA_ and P_dil_, the two primary parameters which define aerobic and substrate metabolism and allow the calculation of oxygen consumption in the sample immediately before metabolic arrest. The three remaining parameters T_trans_, P_anap,_ and J_exch_ are not our target parameters and cannot be determined with great precision. However, these auxiliary parameters are taken into account to evaluate their effect on the estimation of the primary parameters. To improve the estimation and to help avoid local minima in parameter space with physiologically implausible values of the auxiliary parameters, *a priori* information for such parameters (θ_i_) can be directly entered into the cost function by adding a prior term to the cost function in Equation 1 for the deviation from a certain reference value θ_i_^*^

(2) $$\mathrm{prior}\left(\theta_{i}\right)=\frac{1}{2}{\left(\frac{\ln\theta_{i}-\ln\theta_{i}^{*}}{\sigma_{\ln\theta_{i}}}\right)}^{2}$$

where $\sigma_{\ln\theta_{i}}$ is the standard deviation for the auxiliary parameter in log-space. The advantage of logarithmic parameters is that the parameter values with a Gaussian prior distribution are positive and dimensionless. Note that the prior probability in Equation 2 does not include the normalization factor for the lognormal distribution of $\frac{1}{\sigma \sqrt{2\pi }}$. Normalization was not necessary because our method applied the Metropolis-Hastings algorithm which uses the ratios of probabilities.

In previous studies, the value of T_trans_ had been estimated to be 0.202 min which is compatible with the time constant of the enrichment of acetyl-CoA with radioactive label [6,8]. We constrain T_trans_ around this prior value with $\sigma_{\ln\theta_{i}}$ set to 0.336, a high value used in a previous study for unreported experimental errors [21]. This is slightly higher than the value for the standard deviation of these parameters determined in simulations by Binsl et al. [8]. The central 95% region of the prior for T_trans_ lies between 0.202/1.96 and 0.202 *1.96 min, since $\sigma_{\ln\theta_{i}}$ = 0.336 = 1/4*(ln(θ_i_ * 1.96)-ln(θ_i_/1.96)), (see [21]).

The accurate quantification of the exchange flux J_exch_ between α-amino and α-keto acids was found to be challenging [2,22]. A previous analysis of the model used in this study revealed a low sensitivity of estimations of J_TCA_ to variations of J_exch_ in the physiological range from 5–60 μmol/(min*gdw) [6]. Reported values of exchange flux in the literature vary substantially. Some report high values for the exchange flux (e.g. 13-fold the flux of J_TCA_[20]). Several other studies report J_exch_ to be approximately equal to J_TCA_[23,24]. To address this issue, we set a prior on J_exch_ relative to the value of J_TCA_. Instead of calculating the prior cost directly from J_exch_, it is therefore determined by entering the ratio θ_i_ = J_exch_/ J_TCA_ into Equation 2. The reference value θ_i_^*^ for the ratio is set to 1, based on values for J_exch_/ J_TCA_ reported by Nuutinen et al. [23] and Yu et al. [24].

Because of the large spread of values found in the literature (see above), we assumed a high standard deviation for the ratio J_exch_/ J_TCA_ and set $\sigma_{\ln\theta_{i}}$ to 1/4*(ln(θ_i_ * 15)-ln(θ_i_/15)) = 1.345, with θ_i_ = 1. It is thereby ensured that J_exch_ lies with 95% probability between J_TCA_/15 and J_TCA_*15.

**Figure 2:**
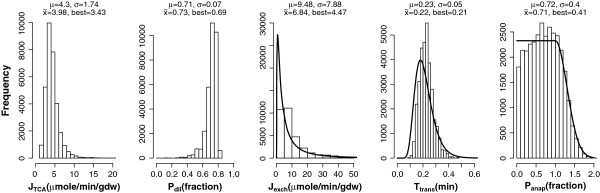
**Posterior distributions for the parameter ensemble (corresponding to 35000 parameter sets) for one tissue sample of the control group.** The probability density functions of the priors for the auxiliary parameters J_exch_, T_trans_ and P_anap_ are plotted with solid lines. On top of each plot, ensemble mean, standard deviation, median (x̃) and best fit value are reported. Note that the probability density functions are scaled to the observed frequencies on the histogram.

For the parameter P_anap_, the anaplerotic flux relative to the TCA cycle flux, most of the values found in literature were smaller than 1 and the highest experimental value found was reported to be 1 ± 0.3 [25,26]. Hence the prior cost for P_anap_ was set to be uniform for values of P_anap_ between 0 and 1 combined with a half-normal distribution which had a standard deviation of 0.3 taken from Lloyd et al. [26] for the values above 1:

(3) $$\mathrm{prior}\left(P_{\mathrm{anap}}\right)=\;\left\{\begin{array}{cc} -\ln\left(c1\right), & \left|0\leq P_{\mathrm{anap}}\leq 1\right)\\ -\ln(N\left(\mu =1,\sigma =0.3\right)*c2), & \left|P_{\mathrm{anap}}>1\right) \end{array}\right\}$$

with $c1=1-\frac{0.5}{0.5+\frac{1}{\sqrt{2\pi \sigma^{2}}}}$ and $c2=\frac{1}{0.5+\frac{1}{\sqrt{2\pi \sigma^{2}}}}$. The normalization constants c1 and c2 ensure that the probability density function of the prior is continuous and that its integral is equal to one. N denotes the normal distribution. The probability density functions for prior(T_trans_), prior(J_exch_), and prior(P_anap_) are shown in Figure 2 (solid lines).

### Parameter estimation and sampling of parameter ensembles

In biological models, usually many different combinations of parameters can describe the experimental data [10]. To address this, we decided to not merely rely on a single best-fit of the model parameters to the NMR data for fixed values of the auxiliary parameters, but instead, we systematically generated ensembles of model parameters that fit the data with reasonable precision. This approach clarifies how well the primary parameters are defined by the data despite uncertainty in the NMR intensities and auxiliary parameters. Through the use of an MCMC approach, confidence bounds can be set on the estimated parameter values. Sampling is based on Bayesian inference of a posterior parameter distribution

(4) $$\mathrm{Pr}\left(\theta |D\right)=\mathrm{Pr}\left(D|\theta \right)*\mathrm{Pr}\left(\theta \right)$$

where *Pr*(*D*|*θ*) is the probability of a parameter vector θ to describe the given data *D* and *Pr*(*θ*) is the prior probability of the parameters (see above). The right-hand side of Equation 4 is equal to *e*^− *C*(*θ*)^ where the cost function of Equation 1 (which includes the priors of Equations 2 and 3) is used. The probability functions were not all normalized because this was not necessary for the MCMC procedure which relies on the ratios of probabilities rather than absolute values. Note that the cost function (Equations 1, 2, 3) forms the basis of a probability function (Equation 4) that defined the ensemble of estimated parameter values.

In order to estimate the model parameters and to quantify the uncertainty of the estimated values, we sampled an ensemble of parameter sets which could describe the available NMR data by performing a random walk through the parameter space through the application of the Metropolis-Hastings algorithm. The starting point of the random walk was an optimized set of parameters, which had been obtained by a grid optimization strategy introduced by Binsl et al. [8]. The grid optimization was designed to cope with a shallow basin shaped by the cost function in order to avoid optimization towards local minima. The procedure covered the 5-dimensional parameter space by a grid so as to find the best starting point for optimization. The second phase of optimization starting at this grid point was then performed using the Nelder & Mead simplex algorithm, and in the third phase we used the Metropolis-Hastings algorithm to sample a parameter ensemble with its probability density proportional to a probability function based on the cost function C(θ) of Equation 1 entered in Equation 4.

### Quality criteria for flux estimations in NMR samples

In many of the available *in vivo* samples, NMR peak intensities are low and often below the threshold of observability, i.e. often six or seven of the nine multiplets of glutamate are not discernible from noise and were assigned an intensity of zero. In some of these low intensity samples, Monte Carlo sampling leads to very large ensemble standard deviations of the estimated primary parameters. We excluded such samples which did not yield reliable estimates for J_TCA_. The exclusion criterion was that the standard deviation of J_TCA_ in the posterior parameter ensemble exceeded 10 μmol/(min*gdw).

### Software package FluxEs

The analysis was performed using the R package FluxEs introduced by Binsl et al. [8]. In order to process parameter ensembles, a Monte Carlo module was added to the software. This module uses the AMCMC algorithm implemented within the package spBayes [27,28]. The AMCMC algorithm is a Metropolis-Hastings variant which automatically adapts the proposal step size for the sampled parameters in the random walk. This leads to quicker convergence to a posterior distribution. For the primary parameters, the time constant of the autocorrelation function of the sampled ensemble was calculated in order to inspect whether the algorithm converged to a stationary distribution. For samples with a high autocorrelation time in the primary parameters, we visually inspected the parameter trace.

A single model simulation run takes approximately 0.26 seconds on a computer with 2.26 GHz clock frequency. The grid optimization for a single sample took on average 115 minutes, the subsequent sampling with the adaptive Metropolis-Hastings algorithm took on average 540 minutes per sample.

The calculations for all samples were performed in parallel on the Lisa computer cluster system at SARA Computing and Networking Services (http://www.sara.nl). All code required for the analysis and part of the experimental data can be found in Additional file 2.

## Results

### Monte-Carlo sampling

We estimated the TCA cycle flux from the NMR peaks of glutamate for 347 tissue samples from 38 hearts. Applying the exclusion criterion described above we removed 85 low-quality samples - leaving 262 samples for further analysis. For each sample, an ensemble of 35,000 parameter sets was generated with the Metropolis-Hastings algorithm. Although convergence was not the first criterion for sample rejection, all ensemble estimates with a high autocorrelation time constant were rejected according to the quality criterion.

An example of a parameter ensemble for one single sample of the control group is given in Figure 2. For T_trans_, J_exch,_ and P_anap_, the probability density functions of the prior distributions are plotted together with the histograms of the posterior distributions. The posterior distributions for these auxiliary parameters are very broad and relatively close to their corresponding prior distributions. In this way the MCMC ensemble method allowed defining the uncertainty in the primary parameters taking into account the large spread in auxiliary parameters. Despite the broad distribution of the auxiliary parameters, the estimates for J_TCA_ and P_dil_ form relatively well-defined peaks and their standard deviations are relatively low.

For the primary parameters we can thus provide point estimates for each sample. To determine which measure best reflects the true value of a parameter, we conducted a simulation experiment in which multiple sets of artificial NMR multiplets were generated by model simulation and subsequent addition of Gaussian random measurement noise. The parameters were then re-estimated and we compared the estimates from the best fit after grid optimization (i.e. the fit with the lowest cost function value and therefore the highest likelihood, see Equation 4) and the mean, median, and mode of the Monte Carlo ensemble with the “true” parameter values from the initial simulation. Regarding the primary parameters, the best fit gave the most reliable point estimate. Below, we therefore report the best fit values for the primary parameters.

### Validation by estimation of myocardial oxygen consumption

In order to validate our flux estimation method we compared the LIPSSS estimated myocardial oxygen consumption (MVO_2_, expressed in μmol/(min*gdw)) with independent “gold standard” measurements. The “gold standard” was determined by blood-gas oxygen and blood flow measurements and the LIPSSS estimated oxygen consumption was calculated from the parameter estimates of the model [18]. The MVO_2_ for a single sample is determined from the primary LIPSSS flux parameters by stoichiometric biochemical relations and can be calculated as follows [8,29]:

(5) $$\mathrm{MV}O_{2}^{\mathrm{sample}}=\left(2+P_{\mathrm{dil}}\right)*J_{\mathrm{TCA}}$$

The MVO_2_ determined from blood-gas measurements reflects the oxygen consumption of the entire heart. When averaging the samples taken for LIPSSS measurements to estimate oxygen consumption for the entire heart ($\mathrm{MV}O_{2}^{\mathrm{heart}}$), individual sample sizes were taken into account. As in Binsl et al., the contributions of the individual samples were weighted by the dry weight w^sample^ for each sample [8].

(6) $$\mathrm{MV}O_{2}^{\mathrm{heart}}=\frac{\sum w^{\mathrm{sample}}*\mathrm{MV}O_{2}^{\mathrm{sample}}}{\sum w^{\mathrm{sample}}}$$

**Figure 3:**
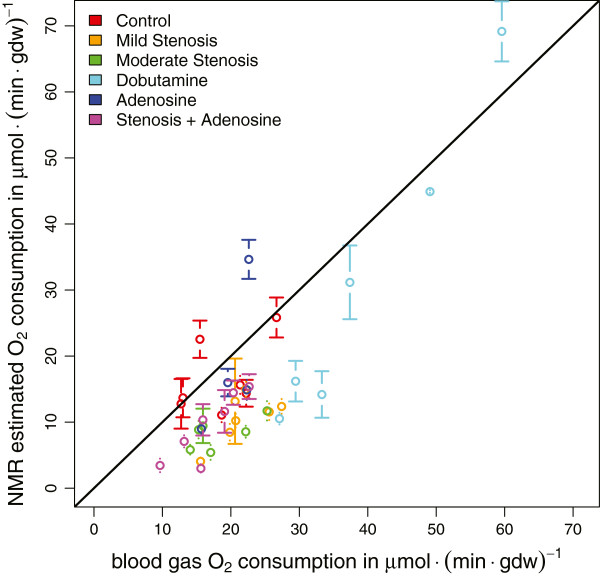
**“Gold standard” oxygen consumption (x-axis) calculated from blood gas and blood flow measurements versus oxygen consumption calculated from the parameter estimates derived with the LIPSSS method (y- axis).** Each data point corresponds to one heart. The line of identity is plotted in black. Error bars correspond to the standard error of the mean of the oxygen consumption based on NMR measurements over all samples taken from one heart. Note that the error for blood-gas measurements using radioactive microspheres to measure local blood flow is estimated to be about 9% accounting for measurement error, spatial and temporal variation.

For all six experimental groups, the comparison of MVO_2_ estimated with the LIPSSS method (from the model parameters P_dil_ and J_TCA_) with the “gold standard” oxygen measurements is shown in Figure 3. One heart from the stenosis + adenosine group was excluded from the analysis since none of its samples satisfied the quality criterion.

For all groups, LIPSSS MVO_2_ correlated with blood-gas MVO_2_ relatively well. For the control group oxygen consumption measured by the two methods corresponded, but for the ischemic conditions (stenosis with and without adenosine), oxygen consumption tended to be lower for the LIPSSS method. We calculated Pearson correlation coefficients of 0.49 for control (n = 7, p = 0.26), 0.69 for mild stenosis (n = 7, p = 0.09), 0.66 for moderate stenosis (n = 6, p = 0.15), 0.99 for dobutamine (n = 6, p = 0.0003), 0.71 for adenosine (n = 4, p = 0.29), and 0.87 for the stenosis + adenosine group (n = 7, p = 0.01). The Pearson correlation for all groups combined was 0.85 (n = 37, p < 10^-10^). The dobutamine group showed higher oxygen consumption than the other groups reflecting the increased cardiac work load. It is important to note that the small tissue biopsies used in the LIPSSS experiment only covered a relatively small cardiac region, in contrast to the physiological blood-gas measurements which covered the entire left ventricle. Furthermore, the estimation of MVO_2_ from the parameters of the TCA cycle model only reflects myocardial oxygen consumption linked to the TCA cycle flux, disregarding other oxygen consuming reactions which were covered by the blood-gas measurements. The oxygen consumption measurements in a small ischemic region dependent on a constricted coronary artery would be very difficult to obtain with classic blood-gas measurements.

### Estimation of TCA cycle fluxes

**Figure 4:**
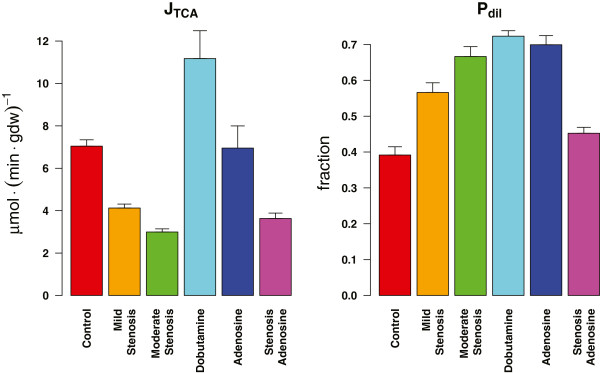
**Estimates for the primary model parameters for all experimental groups.** J_TCA_ and P_dil_ denote the overall TCA cycle flux and the dilution fraction of labeled acetate entering the TCA cycle due to unlabeled endogenous substrates such as glucose and fatty acids, respectively. Estimates were first averaged for each heart and then all hearts were averaged for each group. The error bars represent the standard error of the mean (SEM) of the estimates of all hearts in one experimental group.

LIPSSS-based estimates for the primary model parameters under all experimental conditions are shown in Figure 4. Estimates for J_TCA_ in the control group averaged 7.04 ± 0.79 (mean ± SEM) μmol/(min*gdw). For mild and moderate constriction of the coronary vessels, we estimated J_TCA_ to be 4.12 ± 0.49 and 2.99 ± 0.36 μmol/(min*gdw), respectively.

Dobutamine infusion, which stimulates cardiac contraction, leads to a high average J_TCA_ estimate of 11.18 ± 1.31 μmol/(min*gdw) of tissue. Estimations for the adenosine group show no difference with the control condition. The TCA cycle flux in the stenosis + adenosine group is in between the mild and moderate stenosis condition.

The relative contribution to the TCA cycle flux of substrates other than labeled acetate, i.e. P_dil_ is higher in all experimental groups compared with the baseline condition (see Figure 4). Low fractional acetate usage, i.e. high dilution has been previously documented in experiments with dobutamine [30].

The estimations of T_trans_ for all the groups did not differ substantially from the prior value of 0.202 minutes (data not shown). Ensembles for the auxiliary parameters J_exch_ and P_anap_ show large standard deviations. This indicates that these parameters cannot be estimated properly from the NMR data. Indeed, the experimental protocol was optimized to estimate the primary parameters, disregarding the auxiliary parameters. Nevertheless the effect of the potential spread in these auxiliary parameters on the uncertainty limits of the primary parameters was taken into account. Estimations of the auxiliary model parameters are described in supplemental file 3.

## Discussion

The fluxes of biochemical reactions linked to cardiac energy metabolism are of significant interest. Here we investigated a computational method to quantify fluxes in the TCA cycle using NMR data from ^13^C labeling experiments in porcine hearts. We took measurement error in the data and uncertainty of model parameters directly into account. To test the method, distinct ^13^C labeling patterns (isotopomers) in glutamate were measured under six different cardiac stress and control conditions. The data were analyzed with a detailed model of carbon transitions in the TCA cycle and two primary flux parameters of interest (reflecting total aerobic metabolism and uptake of the labeled substrate) were estimated. Possible variation in three auxiliary parameters, taken from experimental literature was included in the application of Bayesian priors. To define the uncertainty in estimated flux parameters from measurement error and uncertainty in prior knowledge, we used an MCMC method. As a result, we were able to derive estimates for the TCA cycle fluxes under various experimental conditions despite the high noise level in the available NMR data. For validation, we compared blood-gas measurements of myocardial oxygen consumption with oxygen consumption calculated from our own parameter estimates. The oxygen consumption estimated with our model correlated with the classic physiological measurements for the whole heart (Figure 3).

However, because the LIPSSS parameter estimates relied on small samples obtained from the heart while the blood gas measurements represented the oxygen consumption for the whole heart, the LIPSSS estimates are expected to deviate from the whole heart measurement. The deviation may have a random component because of the limited tissue sample size, and a systematic component because of functional differences between regions in the heart. The random component is expected because heterogeneity of blood flow and metabolism has been measured in heart muscle [18,31]. A systematic component is expected especially in the stenosis groups, because the LIPSSS NMR measurements are taken from regions with lower oxygen consumption caused by low perfusion. However, it should be noted that this reasoning is incomplete because the blood gas estimation of oxygen consumption takes the local blood flow measured in the stenosed region into account. Nevertheless, systematic differences between the small region and the average for the whole heart may contribute to the deviation from the line of identity (see Figure 3) at low oxygen consumptions.

Additional physiological measurements of oxygen consumption and metabolic fluxes, independent from the stable isotope labeling experiments, are desirable for further validation of our method. Regional rates of oxygen consumption can be measured by measuring oxygen content in small veins [31] with a spectroscopical method in frozen tissue. The latter method is difficult and its validation has been criticized. A further method is the simultaneous determination of myocardial perfusion and oxygen content in small regions of the heart [32]. Oxygen consumption can also be measured using PET and TCA cycle fluxes using in vivo NMR (e.g. [33]). However, these methods mostly have very limited spatial resolution [32] and were in turn subject to rather limited validation themselves. The difficulty in measuring local energy metabolic flux provided motivation to develop our present method in the first place. Despite the limited possibilities, further validation of the LIPSSS method in the future is desirable.

Part of the dataset used here, namely the control and dobutamine group, had been analyzed in a previous study [8]. The estimates of Binsl et al. [8] relied on prior information on the model parameters T_trans_ and P_anap_. The latter parameter, describing anaplerosis relative to the TCA cycle flux was constrained to 6 ± 3% of J_TCA_, based on information from literature studies on isolated hearts. The latter studies however, only accounted for anaplerosis from either propionate [34] or from pyruvate [35,36]. It has been suggested that relative anaplerosis is often underestimated by conventional approaches, including isotopomer analysis or fractional enrichments of carbons in glutamate [37]. Tracer experiments also exist using ^13^C labeled propionate that report the relative anaplerotic flux in rat hearts to be much higher than 6%, e.g. 16% [38] or 29% [19]. Higher relative anaplerotic fluxes were reported during low flow ischemia, reaching 100% [26] and 35% [25]. Higher values have also been reported for hypertrophy [39]. Although our estimates for the parameter P_anap_ in the present study have a relatively low precision, they suggest the possibility that in porcine heart anaplerotic flux *in vivo* is relatively high in contrast to low values often found in isolated hearts (see Additional file 3).

Since three different stenosis conditions were included in the present study, we chose a less constraining Bayesian prior on the parameter P_anap_ which covered a broad range. It is important to note that the Bayesian priors were the same for the analysis of NMR data from all experimental conditions. Despite the use of different choices of priors on the parameters, and although a higher anaplerotic flux was estimated (see Additional file 3), our present estimates for fluxes in the control and dobutamine groups did not differ much from the previous estimates of Binsl et al. [8]. Our estimates for cardiac ischemia induced by coronary stenosis show that the TCA cycle flux decreases whilst the relative anaplerosis increases (see Figure 4 and Additional file 3) which is compatible with existing literature (see references cited above).

Due to the high velocity of the exchange reactions between α-amino and α-keto acids, the determination of J_exch_ using tracer experiments is expected to be practically infeasible [22]. Because of the uncertainty on J_exch_, we decided to evaluate the effect of variation in J_exch_. Values for J_exch_/J_TCA_ found in literature vary between 0.2 and 13 [20,22], but are often around 1 in the heart [24], in contrast to the very high J_exch_/J_TCA_ reported for the human brain [40]. Initial estimations of J_exch_ in our data showed that, particularly in samples with low NMR peak intensity, the simulated isotope enrichment was not very sensitive to J_exch_. Rather than constraining J_exch_ around an absolute value, we chose to set a Bayesian prior relative to J_TCA_. The standard deviation of the prior was set to a very high value, reflecting the high variability of J_exch_/J_TCA_ measurements found in the literature. J_exch_/J_TCA_ estimated with our method ranged from 0.74 (median dobutamine group) to 1.75 (median control group). Weiss et al. reported a decreased absolute exchange flux compared with control conditions during post-ischemic reperfusion in rat hearts [41]. A decrease in J_exch_ during stenosis was estimated in the present study (see Additional file 3).

Literature information on parameter values was incorporated into the analysis as Bayesian priors because of the high noise level in the NMR data. Without using prior information, flux parameters sometimes reach physiologically infeasible regions in parameter space. We investigated the sensitivity of our estimates of the primary parameters to the priors for the auxiliary parameters by re-performing the analysis with doubled prior standard deviations in equations 2 and 3. The estimate for parameter P_dil_ is rather insensitive to changes in the prior standard deviation (absolute difference in the estimated value averaged over all groups is 4.4±4.0%) while estimates of J_TCA_ are more sensitive to alterations in the priors on auxiliary parameters (average absolute difference 20±21%). Especially in the moderate stenosis group, for which the NMR signals are on average very low, many estimates fail to meet the quality criteria if the standard deviation for all three priors simultaneously was made twice as large. This shows that the estimate of J_TCA_ is sensitive to the prior. However, Bayesian priors are necessary to constrain the estimates within reasonable ranges. It is therefore important to emphasize that the prior values and their standard deviations are not arbitrarily chosen. The prior distributions of P_anap_ and J_exch_ are based on experimental data [20,23]–[26] and were given large standard deviations. The prior on T_trans_ is based on previous estimates [6,8] and its standard deviation allows for a broad range. We therefore argue that although constraining parameters in this study was necessary due to the high noise in the data, our framework still allowed to define reasonable point estimates of flux parameters and additionally to define the variability in parameter estimates taking reasonable, sometimes deliberately high, values for the uncertainty of auxiliary parameters into account.

Parametric sensitivity analysis is commonly applied in systems biology [42]. In this investigation, we chose an approach that explored the multidimensional space around a set of best-fit parameters using a random walk with the Metropolis-Hastings algorithm [9,10,21]. The advantage of this method is that it takes into account possible correlations and nonlinear dependencies between the model parameters. Antoniewiecz et al. approached the problem of defining confidence regions for flux estimates by minimizing a sum of squared residuals objective function as a function of the flux value [17]. In their approach, the confidence interval for a flux of interest is derived by setting the flux constant while optimizing all remaining fluxes in the system. This step is repeated for a range of fixed flux values until the objective function value exceeds a predefined confidence limit. The advantage of the MCMC approach to determine confidence regions is that it takes all possible correlations between the fluxes into account, since no flux parameter is fixed during the MCMC sampling.

The challenge in analyzing the data in this study was the high noise level. Up to seven of the nine measured multiplet intensities could sometimes not be detected. Ensemble modeling proved to be a feasible method to separate samples with flux parameters that could be estimated from samples with poor information on the fluxes in the system. This ensemble approach made it possible to identify 262 out of 347 samples that gave useful estimates for the primary parameters. The quality selection of the samples allowed us to use the best-fit parameters from each sample as a point estimate for the primary parameters. The MCMC approach allowed us to define confidence bounds on all estimated parameter values taking their correlations into account. This is a significant advantage compared with previous approaches, where linearized or analytical methods were used to calculate errors on estimated model parameters [5,6,8].

Adding the Monte Carlo ensemble sampling to the LIPSSS framework enables us to estimate the confidence regions of flux parameters in a single sample. The small size of the tissue samples makes it feasible to identify the spatial variation of flux parameters expected because of the known heterogeneity in the tissue. The physiological meaning of our measurements of heterogeneity in metabolism in heart muscle will be addressed in future studies.

## Conclusions

In this study we improved the LIPSSS method in order to quantify metabolic fluxes using stable isotope labeling integrated with mathematical models of carbon transitions: auxiliary information was taken into account in the form of Bayesian priors and emphasis was placed on the uncertainty analysis of the estimated flux parameters. The method was used to quantify TCA cycle fluxes from noisy NMR measurements in porcine hearts under different physiological conditions. Two important metabolic fluxes could be determined in single biopsies taken during animal experiments and confidence regions could be calculated for single samples.

## Competing interests

The authors declare that they have no competing interests.

## Authors’ contributions

DJA, ABJG and JHGMvB designed and conducted the animal experiments. HH, TWB and JHGMvB designed and conceived the *in silico* experiments, HH and TWB performed the *in silico* experiments. HH, DJA, JH and JHGMvB analyzed the data. HH and JHGMvB wrote the manuscript. All authors read and approved the final manuscript.

## Supplementary Material

Additional file 1 **Model equations.** In this supplemental text, we give a detailed description of the computational model and list all model ODEs. Click here for file

Additional file 2 **Code and data.** In this supplemental file we provide all R code and part of the experimental data used to produce the results of this study. The zip file contains a file README.txt which describes all code and data. Click here for file

Additional file 3 **Estimation of auxiliary model parameters.** In this supplemental text, the results of estimating the auxiliary model parameters P_anap_ and J_exch_ are presented and discussed. Click here for file
