# Supplementary material for: Computational estimation of tricarboxylic acid cycle fluxes using noisy NMR data from cardiac biopsies
Source: BMC Syst Biol. 2013 Aug 21;7:82. doi: 10.1186/1752-0509-7-82 (PMC3765389; doi:10.1186/1752-0509-7-82)
Supplement: Additional file 1 — Model equations. In this supplemental text, we give a detailed description of the computational model and list all model ODEs. [file 1752-0509-7-82-S1.pdf]

Supplemental Text: Model Equations

(Computational estimation of Tricarboxylic Acid Cycle fluxes using noisy NMR data from cardiac biopsies)

Hannes Hettling, David J.C. Alders, Jaap Heringa, Thomas W. Binsl, A.B. Johan Groeneveld, and Johannes H.G.M. van Beek

This document gives a detailed description of the model used in this study which was initially described in [1, 2] and modified in [3]. Equations 1-132 (see below) describe the time derivative of each isotopomer for all metabolites in the model. A model assumption is that metabolite contents and fluxes are constant.

The following metabolites are considered in the model: acetate, acetyl-CoA, succinate, citrate, α-ketoglutarate, glutamate, oxaloacetate, and aspartate. Influx from a diluting pool accounts for the dilution of labeled acetate entering the TCA cycle. An anaplerotic pool represents four-carbon metabolites that exchange with TCA cycle intermediates. See Figure 1 in the main text for a scheme of the model.

In the model equations, the labeling state of each metabolite is given by its binary subscript. The subscripts have the length of the number of carbons of the respective metabolites and represent the isotopomers in binary form. For example, the variable Acetate_{00} represents unlabeled acetate, Acetate_{01} and Acetate_{10} represent acetate labeled at the first and second carbon atom, respectively, ams Acetate_{11} stands for fully labelled acetate. Note that the sixth carbon of citrate is not specified, because it leaves in CO_2 before the next pool in the model is reached.

Acetyl-CoA and succinate are implemented as *virtual pools*. Virtual pools are implemented with algebraic rather than differential equations and mix incoming metabolites without any time delay, which approximates mixing of metabolic fluxes in small metabolite pools exhibiting fast turnover rates [3]. An example is the equation for unlabeled Acetyl-CoA ($\text{AcetylCoaVirtual}_{00}$):

$$\frac{d\text{AcetylCoaVirtual}_{00}}{dt} = J_{\text{TCA}} * (1 - P_{\text{dil}}) * \text{Acetate}_{00} + J_{\text{TCA}} * P_{\text{dil}} * \text{DilutingPool}_{00} - J_{\text{TCA}} * \text{AcetylCoaVirtual}_{00}$$

The left-hand term of the differential equation is equal to zero because the virtual pool reaches a steady state immediately. This results in an algebraic equation. The algebraic equations for acetyl-CoA are integrated in the differential equations for citrate (equations 5-36 below), and the algebraic equations for succinate are integrated in the differential equations for oxaloacetate (equations 101-116).

Pool concentrations, taken from [4] and [2], given in $\mu mole * gdw^{-1}$ (dw = dry weight) were as follows: citrate: 1.41, α-ketoglutarate: 0.2, oxaloacetate: 2.5 and aspartate 1.87. The concentration of the glutamate pool varies in this study and was measured for each sample.

It is important to note that in this study, a double labeling experiment is simulated in which $[2-^{13}C]$ acetate is infused followed by $[1,2-^{13}C]$ acetate. Infused acetate is termed Acetate_in. We simulate this protocol by integrating the ODE model in two phases, where the start values for the second simulation are the fractional isotopomer values at the end of the first simulation. Acetate_in_{01} is 0.99 during the first 4 minutes of $[2-^{13}C]$ acetate infusion. Acetate_in_{11} is set to 0.99 between 4 and 5.5 minutes during $[1,2-^{13}C]$ acetate infusion. Due to natural abundance of ^{13}C at 1.1% the rest of the Acetate_in fractional values are not exact at zero but close to zero during the whole simulation (0-5.5 minutes).

References

[1] van Beek JH, Csont T, de Kanter FJ, Bussemaker J: **Simple model analysis of 13C NMR spectra to measure oxygen consumption using frozen tissue samples.** *Advances in Experimental Medicine and Biology* 1998, **454**:475–85, [[http://www.ncbi.nlm.nih.gov/pubmed/9889927]].

[2] van Beek JH, van Mil HG, King RB, de Kanter FJ, Alders DJ, Bussemaker J: **A (13)C NMR double-labeling method to quantitate local myocardial O(2) consumption using frozen tissue samples.** *The American Journal of Physiology* 1999, **277**(4 Pt 2):H1630–40, [[http://www.ncbi.nlm.nih.gov/pubmed/10516204]].

[3] Binsl TW, Alders DJC, Heringa J, Groeneveld ABJ, van Beek JHGM: **Computational quantification of metabolic fluxes from a single isotope snapshot: application to an animal biopsy.** *Bioinformatics (Oxford, England)* 2010, **26**(5):653–60, [[http://bioinformatics.oxfordjournals.org/cgi/content/abstract/26/5/653]].

[4] Alders DJC, Groeneveld ABJ, de Kanter FJJ, van Beek JHGM: **Myocardial O2 consumption in porcine left ventricle is heterogeneously distributed in parallel to heterogeneous O2 delivery.** *American Journal of Physiology. Heart and Circulatory Physiology* 2004, **287**(3):H1353–61, [[http://www.ncbi.nlm.nih.gov/pubmed/15142850]].

Differential Equations

$$\frac{d\text{Acetate}_{00}}{dt} = (\text{Acetate\_in}_{00} - \text{Acetate}_{00})/T_{\text{trans}} \tag{1}$$

$$\frac{d\text{Acetate}_{01}}{dt} = (\text{Acetate\_in}_{01} - \text{Acetate}_{01})/T_{\text{trans}} \tag{2}$$

$$\frac{d\text{Acetate}_{10}}{dt} = (\text{Acetate\_in}_{10} - \text{Acetate}_{10})/T_{\text{trans}} \tag{3}$$

$$\frac{d\text{Acetate}_{11}}{dt} = (\text{Acetate\_in}_{11} - \text{Acetate}_{11})/T_{\text{trans}} \tag{4}$$

$$\frac{d\text{Citrate}_{00000}}{dt} = ((\text{Acetate}_{00} * (J_{\text{TCA}} * (1 - P_{\text{dil}})) + \text{DilutingPool}_{00} * (J_{\text{TCA}} * P_{\text{dil}}))/(J_{\text{TCA}} * (1 - P_{\text{dil}}) + J_{\text{TCA}} * P_{\text{dil}}) * (\text{Oxaloacetate}_{0000} + \text{Oxaloacetate}_{0001}) * J_{\text{TCA}} - J_{\text{TCA}} * \text{Citrate}_{00000})/[\text{Citrate}] \tag{5}$$

$$\frac{d\text{Citrate}_{00001}}{dt} = ((\text{Acetate}_{00} * (J_{\text{TCA}} * (1 - P_{\text{dil}})) + \text{DilutingPool}_{00} * (J_{\text{TCA}} * P_{\text{dil}}))/(J_{\text{TCA}} * (1 - P_{\text{dil}}) + J_{\text{TCA}} * P_{\text{dil}}) * (\text{Oxaloacetate}_{1000} + \text{Oxaloacetate}_{1001}) * J_{\text{TCA}} - J_{\text{TCA}} * \text{Citrate}_{00001})/[\text{Citrate}] \tag{6}$$

$$\frac{d\text{Citrate}_{00010}}{dt} = ((\text{Acetate}_{00} * (J_{\text{TCA}} * (1 - P_{\text{dil}})) + \text{DilutingPool}_{00} * (J_{\text{TCA}} * P_{\text{dil}}))/(J_{\text{TCA}} * (1 - P_{\text{dil}}) + J_{\text{TCA}} * P_{\text{dil}}) * (\text{Oxaloacetate}_{0100} + \text{Oxaloacetate}_{0101}) * J_{\text{TCA}} - J_{\text{TCA}} * \text{Citrate}_{00010})/[\text{Citrate}] \tag{7}$$

$$\frac{d\text{Citrate}_{00011}}{dt} = ((\text{Acetate}_{00} * (J_{\text{TCA}} * (1 - P_{\text{dil}})) + \text{DilutingPool}_{00} * (J_{\text{TCA}} * P_{\text{dil}}))/(J_{\text{TCA}} * (1 - P_{\text{dil}}) + J_{\text{TCA}} * P_{\text{dil}}) * (\text{Oxaloacetate}_{1100} + \text{Oxaloacetate}_{1101}) * J_{\text{TCA}} - J_{\text{TCA}} * \text{Citrate}_{00011})/[\text{Citrate}] \tag{8}$$

$$\frac{d\text{Citrate}_{00100}}{dt} = ((\text{Acetate}_{00} * (J_{\text{TCA}} * (1 - P_{\text{dil}})) + \text{DilutingPool}_{00} * (J_{\text{TCA}} * P_{\text{dil}}))/(J_{\text{TCA}} * (1 - P_{\text{dil}}) + J_{\text{TCA}} * P_{\text{dil}}) * (\text{Oxaloacetate}_{0010} + \text{Oxaloacetate}_{0011}) * J_{\text{TCA}} - J_{\text{TCA}} * \text{Citrate}_{00100})/[\text{Citrate}] \tag{9}$$

$$\frac{d\text{Citrate}_{00101}}{dt} = ((\text{Acetate}_{00} * (J_{\text{TCA}} * (1 - P_{\text{dil}})) + \text{DilutingPool}_{00} * (J_{\text{TCA}} * P_{\text{dil}}))/(J_{\text{TCA}} * (1 - P_{\text{dil}}) + J_{\text{TCA}} * P_{\text{dil}}) * (\text{Oxaloacetate}_{1010} + \text{Oxaloacetate}_{1011}) * J_{\text{TCA}} - J_{\text{TCA}} * \text{Citrate}_{00101})/[\text{Citrate}] \tag{10}$$

$$\frac{d\text{Citrate}_{00110}}{dt} = ((\text{Acetate}_{00} * (J_{\text{TCA}} * (1 - P_{\text{dil}})) + \text{DilutingPool}_{00} * (J_{\text{TCA}} * P_{\text{dil}}))/(J_{\text{TCA}} * (1 - P_{\text{dil}}) + J_{\text{TCA}} * P_{\text{dil}}) * (\text{Oxaloacetate}_{0110} + \text{Oxaloacetate}_{0111}) * J_{\text{TCA}} - J_{\text{TCA}} * \text{Citrate}_{00110})/[\text{Citrate}] \tag{11}$$

$$\frac{d\text{Citrate}_{00111}}{dt} = ((\text{Acetate}_{00} * (J_{\text{TCA}} * (1 - P_{\text{dil}})) + \text{DilutingPool}_{00} * (J_{\text{TCA}} * P_{\text{dil}}))/(J_{\text{TCA}} * (1 - P_{\text{dil}}) + J_{\text{TCA}} * P_{\text{dil}}) * (\text{Oxaloacetate}_{1110} + \text{Oxaloacetate}_{1111}) * J_{\text{TCA}} - J_{\text{TCA}} * \text{Citrate}_{00111})/[\text{Citrate}] \tag{12}$$

$$\frac{d\text{Citrate}_{01000}}{dt} = ((\text{Acetate}_{10} * (J_{\text{TCA}} * (1 - P_{\text{dil}})) + \text{DilutingPool}_{10} * (J_{\text{TCA}} * P_{\text{dil}}))/(J_{\text{TCA}} * (1 - P_{\text{dil}}) + J_{\text{TCA}} * P_{\text{dil}}) * (\text{Oxaloacetate}_{0000} + \text{Oxaloacetate}_{0001}) * J_{\text{TCA}} - J_{\text{TCA}} * \text{Citrate}_{01000})/[\text{Citrate}] \tag{13}$$

$$\frac{d\text{Citrate}_{01001}}{dt} = ((\text{Acetate}_{10} * (J_{\text{TCA}} * (1 - P_{\text{dil}})) + \text{DilutingPool}_{10} * (J_{\text{TCA}} * P_{\text{dil}}))/(J_{\text{TCA}} * (1 - P_{\text{dil}}) + J_{\text{TCA}} * P_{\text{dil}}) * (\text{Oxaloacetate}_{1000} + \text{Oxaloacetate}_{1001}) * J_{\text{TCA}} - J_{\text{TCA}} * \text{Citrate}_{01001})/[\text{Citrate}] \tag{14}$$

$$\frac{d\text{Citrate}_{01010}}{dt} = ((\text{Acetate}_{10} * (J_{\text{TCA}} * (1 - P_{\text{dil}})) + \text{DilutingPool}_{10} * (J_{\text{TCA}} * P_{\text{dil}}))/(J_{\text{TCA}} * (1 - P_{\text{dil}}) + J_{\text{TCA}} * P_{\text{dil}}) * (\text{Oxaloacetate}_{0100} + \text{Oxaloacetate}_{0101}) * J_{\text{TCA}} - J_{\text{TCA}} * \text{Citrate}_{01010})/[\text{Citrate}] \tag{15}$$

$$\frac{d\text{Citrate}_{01011}}{dt} = ((\text{Acetate}_{10} * (J_{\text{TCA}} * (1 - P_{\text{dil}})) + \text{DilutingPool}_{10} * (J_{\text{TCA}} * P_{\text{dil}}))/(J_{\text{TCA}} * (1 - P_{\text{dil}}) + J_{\text{TCA}} * P_{\text{dil}}) * (\text{Oxaloacetate}_{1100} + \text{Oxaloacetate}_{1101}) * J_{\text{TCA}} - J_{\text{TCA}} * \text{Citrate}_{01011})/[\text{Citrate}] \tag{16}$$

$$\frac{d\text{Citrate}_{01100}}{dt} = ((\text{Acetate}_{10} * (J_{\text{TCA}} * (1 - P_{\text{dil}})) + \text{DilutingPool}_{10} * (J_{\text{TCA}} * P_{\text{dil}}))/(J_{\text{TCA}} * (1 - P_{\text{dil}}) + J_{\text{TCA}} * P_{\text{dil}}) * (\text{Oxaloacetate}_{0010} + \text{Oxaloacetate}_{0011}) * J_{\text{TCA}} - J_{\text{TCA}} * \text{Citrate}_{01100})/[\text{Citrate}] \tag{17}$$

$$\frac{d\text{Citrate}_{01101}}{dt} = ((\text{Acetate}_{10} * (J_{\text{TCA}} * (1 - P_{\text{dil}})) + \text{DilutingPool}_{10} * (J_{\text{TCA}} * P_{\text{dil}}))/(J_{\text{TCA}} * (1 - P_{\text{dil}}) + J_{\text{TCA}} * P_{\text{dil}}) * (\text{Oxaloacetate}_{1010} + \text{Oxaloacetate}_{1011}) * J_{\text{TCA}} - J_{\text{TCA}} * \text{Citrate}_{01101})/[\text{Citrate}] \tag{18}$$

$$\frac{d\text{Citrate}_{01110}}{dt} = ((\text{Acetate}_{10} * (J_{\text{TCA}} * (1 - P_{\text{dil}})) + \text{DilutingPool}_{10} * (J_{\text{TCA}} * P_{\text{dil}}))/(J_{\text{TCA}} * (1 - P_{\text{dil}}) + J_{\text{TCA}} * P_{\text{dil}}) * (\text{Oxaloacetate}_{0110} + \text{Oxaloacetate}_{0111}) * J_{\text{TCA}} - J_{\text{TCA}} * \text{Citrate}_{01110})/[\text{Citrate}] \tag{19}$$

$$\frac{d\text{Citrate}_{01111}}{dt} = ((\text{Acetate}_{10} * (J_{\text{TCA}} * (1 - P_{\text{dil}})) + \text{DilutingPool}_{10} * (J_{\text{TCA}} * P_{\text{dil}}))/(J_{\text{TCA}} * (1 - P_{\text{dil}}) + J_{\text{TCA}} * P_{\text{dil}}) * (\text{Oxaloacetate}_{1110} + \text{Oxaloacetate}_{1111}) * J_{\text{TCA}} - J_{\text{TCA}} * \text{Citrate}_{01111})/[\text{Citrate}] \tag{20}$$

$$\frac{d\text{Citrate}_{10000}}{dt} = ((\text{Acetate}_{01} * (J_{\text{TCA}} * (1 - P_{\text{dil}})) + \text{DilutingPool}_{01} * (J_{\text{TCA}} * P_{\text{dil}}))/(J_{\text{TCA}} * (1 - P_{\text{dil}}) + J_{\text{TCA}} * P_{\text{dil}}) * (\text{Oxaloacetate}_{0000} + \text{Oxaloacetate}_{0001}) * J_{\text{TCA}} - J_{\text{TCA}} * \text{Citrate}_{10000})/[\text{Citrate}] \tag{21}$$

$$\frac{d\text{Citrate}_{10001}}{dt} = ((\text{Acetate}_{01} * (J_{\text{TCA}} * (1 - P_{\text{dil}})) + \text{DilutingPool}_{01} * (J_{\text{TCA}} * P_{\text{dil}}))/(J_{\text{TCA}} * (1 - P_{\text{dil}}) + J_{\text{TCA}} * P_{\text{dil}}) * (\text{Oxaloacetate}_{1000} + \text{Oxaloacetate}_{1001}) * J_{\text{TCA}} - J_{\text{TCA}} * \text{Citrate}_{10001})/[\text{Citrate}] \tag{22}$$

$$\frac{d\text{Citrate}_{10010}}{dt} = ((\text{Acetate}_{01} * (J_{\text{TCA}} * (1 - P_{\text{dil}})) + \text{DilutingPool}_{01} * (J_{\text{TCA}} * P_{\text{dil}}))/(J_{\text{TCA}} * (1 - P_{\text{dil}}) + J_{\text{TCA}} * P_{\text{dil}}) * (\text{Oxaloacetate}_{0100} + \text{Oxaloacetate}_{0101}) * J_{\text{TCA}} - J_{\text{TCA}} * \text{Citrate}_{10010})/[\text{Citrate}] \tag{23}$$

$$\frac{d\text{Citrate}_{10011}}{dt} = ((\text{Acetate}_{01} * (J_{\text{TCA}} * (1 - P_{\text{dil}})) + \text{DilutingPool}_{01} * (J_{\text{TCA}} * P_{\text{dil}}))/(J_{\text{TCA}} * (1 - P_{\text{dil}}) + J_{\text{TCA}} * P_{\text{dil}}) * (\text{Oxaloacetate}_{1100} + \text{Oxaloacetate}_{1101}) * J_{\text{TCA}} - J_{\text{TCA}} * \text{Citrate}_{10011})/[\text{Citrate}] \tag{24}$$

$$\frac{d\text{Citrate}_{10100}}{dt} = ((\text{Acetate}_{01} * (J_{\text{TCA}} * (1 - P_{\text{dil}})) + \text{DilutingPool}_{01} * (J_{\text{TCA}} * P_{\text{dil}}))/(J_{\text{TCA}} * (1 - P_{\text{dil}}) + J_{\text{TCA}} * P_{\text{dil}}) * (\text{Oxaloacetate}_{0010} + \text{Oxaloacetate}_{0011}) * J_{\text{TCA}} - J_{\text{TCA}} * \text{Citrate}_{10100})/[\text{Citrate}] \tag{25}$$

$$\frac{d\text{Citrate}_{10101}}{dt} = ((\text{Acetate}_{01} * (J_{\text{TCA}} * (1 - P_{\text{dil}})) + \text{DilutingPool}_{01} * (J_{\text{TCA}} * P_{\text{dil}}))/(J_{\text{TCA}} * (1 - P_{\text{dil}}) + J_{\text{TCA}} * P_{\text{dil}}) * (\text{Oxaloacetate}_{1010} + \text{Oxaloacetate}_{1011}) * J_{\text{TCA}} - J_{\text{TCA}} * \text{Citrate}_{10101})/[\text{Citrate}] \tag{26}$$

$$\frac{d\text{Citrate}_{10110}}{dt} = ((\text{Acetate}_{01} * (J_{\text{TCA}} * (1 - P_{\text{dil}})) + \text{DilutingPool}_{01} * (J_{\text{TCA}} * P_{\text{dil}}))/(J_{\text{TCA}} * (1 - P_{\text{dil}}) + J_{\text{TCA}} * P_{\text{dil}}) * (\text{Oxaloacetate}_{0110} + \text{Oxaloacetate}_{0111}) * J_{\text{TCA}} - J_{\text{TCA}} * \text{Citrate}_{10110})/[\text{Citrate}] \tag{27}$$

$$\frac{d\text{Citrate}_{10111}}{dt} = ((\text{Acetate}_{01} * (J_{\text{TCA}} * (1 - P_{\text{dil}})) + \text{DilutingPool}_{01} * (J_{\text{TCA}} * P_{\text{dil}}))/(J_{\text{TCA}} * (1 - P_{\text{dil}}) + J_{\text{TCA}} * P_{\text{dil}}) * (\text{Oxaloacetate}_{1110} + \text{Oxaloacetate}_{1111}) * J_{\text{TCA}} - J_{\text{TCA}} * \text{Citrate}_{10111})/[\text{Citrate}] \tag{28}$$

$$\frac{d\text{Citrate}_{11000}}{dt} = ((\text{Acetate}_{11} * (J_{\text{TCA}} * (1 - P_{\text{dil}})) + \text{DilutingPool}_{11} * (J_{\text{TCA}} * P_{\text{dil}}))/(J_{\text{TCA}} * (1 - P_{\text{dil}}) + J_{\text{TCA}} * P_{\text{dil}}) * (\text{Oxaloacetate}_{0000} + \text{Oxaloacetate}_{0001}) * J_{\text{TCA}} - J_{\text{TCA}} * \text{Citrate}_{11000})/[\text{Citrate}] \tag{29}$$

$$\frac{d\text{Citrate}_{11001}}{dt} = ((\text{Acetate}_{11} * (J_{\text{TCA}} * (1 - P_{\text{dil}})) + \text{DilutingPool}_{11} * (J_{\text{TCA}} * P_{\text{dil}}))/(J_{\text{TCA}} * (1 - P_{\text{dil}}) + J_{\text{TCA}} * P_{\text{dil}}) * (\text{Oxaloacetate}_{1000} + \text{Oxaloacetate}_{1001}) * J_{\text{TCA}} - J_{\text{TCA}} * \text{Citrate}_{11001})/[\text{Citrate}] \tag{30}$$

$$\frac{d\text{Citrate}_{11010}}{dt} = ((\text{Acetate}_{11} * (J_{\text{TCA}} * (1 - P_{\text{dil}})) + \text{DilutingPool}_{11} * (J_{\text{TCA}} * P_{\text{dil}}))/(J_{\text{TCA}} * (1 - P_{\text{dil}}) + J_{\text{TCA}} * P_{\text{dil}}) * (\text{Oxaloacetate}_{0100} + \text{Oxaloacetate}_{0101}) * J_{\text{TCA}} - J_{\text{TCA}} * \text{Citrate}_{11010})/[\text{Citrate}] \tag{31}$$

$$\frac{d\text{Citrate}_{11011}}{dt} = ((\text{Acetate}_{11} * (J_{\text{TCA}} * (1 - P_{\text{dil}})) + \text{DilutingPool}_{11} * (J_{\text{TCA}} * P_{\text{dil}}))/(J_{\text{TCA}} * (1 - P_{\text{dil}}) + J_{\text{TCA}} * P_{\text{dil}}) * (\text{Oxaloacetate}_{1100} + \text{Oxaloacetate}_{1101}) * J_{\text{TCA}} - J_{\text{TCA}} * \text{Citrate}_{11011})/[\text{Citrate}] \tag{32}$$

$$\frac{d\text{Citrate}_{11100}}{dt} = ((\text{Acetate}_{11} * (J_{\text{TCA}} * (1 - P_{\text{dil}})) + \text{DilutingPool}_{11} * (J_{\text{TCA}} * P_{\text{dil}}))/(J_{\text{TCA}} * (1 - P_{\text{dil}}) + J_{\text{TCA}} * P_{\text{dil}}) * (\text{Oxaloacetate}_{0010} + \text{Oxaloacetate}_{0011}) * J_{\text{TCA}} - J_{\text{TCA}} * \text{Citrate}_{11100})/[\text{Citrate}] \tag{33}$$

$$\frac{d\text{Citrate}_{11101}}{dt} = ((\text{Acetate}_{11} * (J_{\text{TCA}} * (1 - P_{\text{dil}})) + \text{DilutingPool}_{11} * (J_{\text{TCA}} * P_{\text{dil}}))/(J_{\text{TCA}} * (1 - P_{\text{dil}}) + J_{\text{TCA}} * P_{\text{dil}}) * (\text{Oxaloacetate}_{1010} + \text{Oxaloacetate}_{1011}) * J_{\text{TCA}} - J_{\text{TCA}} * \text{Citrate}_{11101})/[\text{Citrate}] \tag{34}$$

$$\frac{d\text{Citrate}_{11110}}{dt} = ((\text{Acetate}_{11} * (J_{\text{TCA}} * (1 - P_{\text{dil}})) + \text{DilutingPool}_{11} * (J_{\text{TCA}} * P_{\text{dil}}))/(J_{\text{TCA}} * (1 - P_{\text{dil}}) + J_{\text{TCA}} * P_{\text{dil}}) * (\text{Oxaloacetate}_{0110} + \text{Oxaloacetate}_{0111}) * J_{\text{TCA}} - J_{\text{TCA}} * \text{Citrate}_{11110})/[\text{Citrate}] \tag{35}$$

$$\frac{d\text{Citrate}_{11111}}{dt} = ((\text{Acetate}_{11} * (J_{\text{TCA}} * (1 - P_{\text{dil}})) + \text{DilutingPool}_{11} * (J_{\text{TCA}} * P_{\text{dil}}))/(J_{\text{TCA}} * (1 - P_{\text{dil}}) + J_{\text{TCA}} * P_{\text{dil}}) * (\text{Oxaloacetate}_{1110} + \text{Oxaloacetate}_{1111}) * J_{\text{TCA}} - J_{\text{TCA}} * \text{Citrate}_{11111})/[\text{Citrate}] \tag{36}$$

$$\frac{d\alpha\text{Ketoglutarate}_{00000}}{dt} = (\text{Citrate}_{00000} * J_{\text{TCA}} + \text{Glutamate}_{00000} * J_{\text{exch}} - (J_{\text{TCA}} + J_{\text{exch}}) * \alpha\text{Ketoglutarate}_{00000})/[\alpha\text{Ketoglutarate}] \tag{37}$$

$$\frac{d\alpha\text{Ketoglutarate}_{00001}}{dt} = (\text{Citrate}_{00001} * J_{\text{TCA}} + \text{Glutamate}_{00001} * J_{\text{exch}} - (J_{\text{TCA}} + J_{\text{exch}}) * \alpha\text{Ketoglutarate}_{00001})/[\alpha\text{Ketoglutarate}] \tag{38}$$

$$\frac{d\alpha\text{Ketoglutarate}_{00010}}{dt} = (\text{Citrate}_{00010} * J_{\text{TCA}} + \text{Glutamate}_{00010} * J_{\text{exch}} - (J_{\text{TCA}} + J_{\text{exch}}) * \alpha\text{Ketoglutarate}_{00010})/[\alpha\text{Ketoglutarate}] \tag{39}$$

$$\frac{d\alpha\text{Ketoglutarate}_{00011}}{dt} = (\text{Citrate}_{00011} * J_{\text{TCA}} + \text{Glutamate}_{00011} * J_{\text{exch}} - (J_{\text{TCA}} + J_{\text{exch}}) * \alpha\text{Ketoglutarate}_{00011})/[\alpha\text{Ketoglutarate}] \tag{40}$$

$$\frac{d\alpha\text{Ketoglutarate}_{00100}}{dt} = (\text{Citrate}_{00100} * J_{\text{TCA}} + \text{Glutamate}_{00100} * J_{\text{exch}} - (J_{\text{TCA}} + J_{\text{exch}}) * \alpha\text{Ketoglutarate}_{00100})/[\alpha\text{Ketoglutarate}] \tag{41}$$

$$\frac{d\alpha\text{Ketoglutarate}_{00101}}{dt} = (\text{Citrate}_{00101} * J_{\text{TCA}} + \text{Glutamate}_{00101} * J_{\text{exch}} - (J_{\text{TCA}} + J_{\text{exch}}) * \alpha\text{Ketoglutarate}_{00101})/[\alpha\text{Ketoglutarate}] \tag{42}$$

$$\frac{d\alpha\text{Ketoglutarate}_{00110}}{dt} = (\text{Citrate}_{00110} * J_{\text{TCA}} + \text{Glutamate}_{00110} * J_{\text{exch}} - (J_{\text{TCA}} + J_{\text{exch}}) * \alpha\text{Ketoglutarate}_{00110})/[\alpha\text{Ketoglutarate}] \tag{43}$$

$$\frac{d\alpha\text{Ketoglutarate}_{00111}}{dt} = (\text{Citrate}_{00111} * J_{\text{TCA}} + \text{Glutamate}_{00111} * J_{\text{exch}} - (J_{\text{TCA}} + J_{\text{exch}}) * \alpha\text{Ketoglutarate}_{00111})/[\alpha\text{Ketoglutarate}] \tag{44}$$

$$\frac{d\alpha\text{Ketoglutarate}_{01000}}{dt} = (\text{Citrate}_{01000} * J_{\text{TCA}} + \text{Glutamate}_{01000} * J_{\text{exch}} - (J_{\text{TCA}} + J_{\text{exch}}) * \alpha\text{Ketoglutarate}_{01000})/[\alpha\text{Ketoglutarate}] \tag{45}$$

$$\frac{d\alpha\text{Ketoglutarate}_{01001}}{dt} = (\text{Citrate}_{01001} * J_{\text{TCA}} + \text{Glutamate}_{01001} * J_{\text{exch}} - (J_{\text{TCA}} + J_{\text{exch}}) * \alpha\text{Ketoglutarate}_{01001})/[\alpha\text{Ketoglutarate}] \tag{46}$$

$$\frac{d\alpha\text{Ketoglutarate}_{01010}}{dt} = (\text{Citrate}_{01010} * J_{\text{TCA}} + \text{Glutamate}_{01010} * J_{\text{exch}} - (J_{\text{TCA}} + J_{\text{exch}}) * \alpha\text{Ketoglutarate}_{01010})/[\alpha\text{Ketoglutarate}] \quad (47)$$

$$\frac{d\alpha\text{Ketoglutarate}_{01011}}{dt} = (\text{Citrate}_{01011} * J_{\text{TCA}} + \text{Glutamate}_{01011} * J_{\text{exch}} - (J_{\text{TCA}} + J_{\text{exch}}) * \alpha\text{Ketoglutarate}_{01011})/[\alpha\text{Ketoglutarate}] \quad (48)$$

$$\frac{d\alpha\text{Ketoglutarate}_{01100}}{dt} = (\text{Citrate}_{01100} * J_{\text{TCA}} + \text{Glutamate}_{01100} * J_{\text{exch}} - (J_{\text{TCA}} + J_{\text{exch}}) * \alpha\text{Ketoglutarate}_{01100})/[\alpha\text{Ketoglutarate}] \quad (49)$$

$$\frac{d\alpha\text{Ketoglutarate}_{01101}}{dt} = (\text{Citrate}_{01101} * J_{\text{TCA}} + \text{Glutamate}_{01101} * J_{\text{exch}} - (J_{\text{TCA}} + J_{\text{exch}}) * \alpha\text{Ketoglutarate}_{01101})/[\alpha\text{Ketoglutarate}] \quad (50)$$

$$\frac{d\alpha\text{Ketoglutarate}_{01110}}{dt} = (\text{Citrate}_{01110} * J_{\text{TCA}} + \text{Glutamate}_{01110} * J_{\text{exch}} - (J_{\text{TCA}} + J_{\text{exch}}) * \alpha\text{Ketoglutarate}_{01110})/[\alpha\text{Ketoglutarate}] \quad (51)$$

$$\frac{d\alpha\text{Ketoglutarate}_{01111}}{dt} = (\text{Citrate}_{01111} * J_{\text{TCA}} + \text{Glutamate}_{01111} * J_{\text{exch}} - (J_{\text{TCA}} + J_{\text{exch}}) * \alpha\text{Ketoglutarate}_{01111})/[\alpha\text{Ketoglutarate}] \quad (52)$$

$$\frac{d\alpha\text{Ketoglutarate}_{10000}}{dt} = (\text{Citrate}_{10000} * J_{\text{TCA}} + \text{Glutamate}_{10000} * J_{\text{exch}} - (J_{\text{TCA}} + J_{\text{exch}}) * \alpha\text{Ketoglutarate}_{10000})/[\alpha\text{Ketoglutarate}] \quad (53)$$

$$\frac{d\alpha\text{Ketoglutarate}_{10001}}{dt} = (\text{Citrate}_{10001} * J_{\text{TCA}} + \text{Glutamate}_{10001} * J_{\text{exch}} - (J_{\text{TCA}} + J_{\text{exch}}) * \alpha\text{Ketoglutarate}_{10001})/[\alpha\text{Ketoglutarate}] \quad (54)$$

$$\frac{d\alpha\text{Ketoglutarate}_{10010}}{dt} = (\text{Citrate}_{10010} * J_{\text{TCA}} + \text{Glutamate}_{10010} * J_{\text{exch}} - (J_{\text{TCA}} + J_{\text{exch}}) * \alpha\text{Ketoglutarate}_{10010})/[\alpha\text{Ketoglutarate}] \quad (55)$$

$$\frac{d\alpha\text{Ketoglutarate}_{10011}}{dt} = (\text{Citrate}_{10011} * J_{\text{TCA}} + \text{Glutamate}_{10011} * J_{\text{exch}} - (J_{\text{TCA}} + J_{\text{exch}}) * \alpha\text{Ketoglutarate}_{10011})/[\alpha\text{Ketoglutarate}] \quad (56)$$

$$\frac{d\alpha\text{Ketoglutarate}_{10100}}{dt} = (\text{Citrate}_{10100} * J_{\text{TCA}} + \text{Glutamate}_{10100} * J_{\text{exch}} - (J_{\text{TCA}} + J_{\text{exch}}) * \alpha\text{Ketoglutarate}_{10100})/[\alpha\text{Ketoglutarate}] \quad (57)$$

$$\frac{d\alpha\text{Ketoglutarate}_{10101}}{dt} = (\text{Citrate}_{10101} * J_{\text{TCA}} + \text{Glutamate}_{10101} * J_{\text{exch}} - (J_{\text{TCA}} + J_{\text{exch}}) * \alpha\text{Ketoglutarate}_{10101})/[\alpha\text{Ketoglutarate}] \quad (58)$$

$$\frac{d\alpha\text{Ketoglutarate}_{10110}}{dt} = (\text{Citrate}_{10110} * J_{\text{TCA}} + \text{Glutamate}_{10110} * J_{\text{exch}} - (J_{\text{TCA}} + J_{\text{exch}}) * \alpha\text{Ketoglutarate}_{10110})/[\alpha\text{Ketoglutarate}] \quad (59)$$

$$\frac{d\alpha\text{Ketoglutarate}_{10111}}{dt} = (\text{Citrate}_{10111} * J_{\text{TCA}} + \text{Glutamate}_{10111} * J_{\text{exch}} - (J_{\text{TCA}} + J_{\text{exch}}) * \alpha\text{Ketoglutarate}_{10111})/[\alpha\text{Ketoglutarate}] \quad (60)$$

$$\frac{d\alpha\text{Ketoglutarate}_{11000}}{dt} = (\text{Citrate}_{11000} * J_{\text{TCA}} + \text{Glutamate}_{11000} * J_{\text{exch}} - (J_{\text{TCA}} + J_{\text{exch}}) * \alpha\text{Ketoglutarate}_{11000})/[\alpha\text{Ketoglutarate}] \quad (61)$$

$$\frac{d\alpha\text{Ketoglutarate}_{11001}}{dt} = (\text{Citrate}_{11001} * J_{\text{TCA}} + \text{Glutamate}_{11001} * J_{\text{exch}} - (J_{\text{TCA}} + J_{\text{exch}}) * \alpha\text{Ketoglutarate}_{11001})/[\alpha\text{Ketoglutarate}] \quad (62)$$

$$\frac{d\alpha\text{Ketoglutarate}_{11010}}{dt} = (\text{Citrate}_{11010} * J_{\text{TCA}} + \text{Glutamate}_{11010} * J_{\text{exch}} - (J_{\text{TCA}} + J_{\text{exch}}) * \alpha\text{Ketoglutarate}_{11010})/[\alpha\text{Ketoglutarate}] \quad (63)$$

$$\frac{d\alpha\text{Ketoglutarate}_{11011}}{dt} = (\text{Citrate}_{11011} * J_{\text{TCA}} + \text{Glutamate}_{11011} * J_{\text{exch}} - (J_{\text{TCA}} + J_{\text{exch}}) * \alpha\text{Ketoglutarate}_{11011})/[\alpha\text{Ketoglutarate}] \quad (64)$$

$$\frac{d\alpha\text{Ketoglutarate}_{11100}}{dt} = (\text{Citrate}_{11100} * J_{\text{TCA}} + \text{Glutamate}_{11100} * J_{\text{exch}} - (J_{\text{TCA}} + J_{\text{exch}}) * \alpha\text{Ketoglutarate}_{11100})/[\alpha\text{Ketoglutarate}] \quad (65)$$

$$\frac{d\alpha\text{Ketoglutarate}_{11101}}{dt} = (\text{Citrate}_{11101} * J_{\text{TCA}} + \text{Glutamate}_{11101} * J_{\text{exch}} - (J_{\text{TCA}} + J_{\text{exch}}) * \alpha\text{Ketoglutarate}_{11101})/[\alpha\text{Ketoglutarate}] \quad (66)$$

$$\frac{d\alpha\text{Ketoglutarate}_{11110}}{dt} = (\text{Citrate}_{11110} * J_{\text{TCA}} + \text{Glutamate}_{11110} * J_{\text{exch}} - (J_{\text{TCA}} + J_{\text{exch}}) * \alpha\text{Ketoglutarate}_{11110})/[\alpha\text{Ketoglutarate}] \quad (67)$$

$$\frac{d\alpha\text{Ketoglutarate}_{11111}}{dt} = (\text{Citrate}_{11111} * J_{\text{TCA}} + \text{Glutamate}_{11111} * J_{\text{exch}} - (J_{\text{TCA}} + J_{\text{exch}}) * \alpha\text{Ketoglutarate}_{11111})/[\alpha\text{Ketoglutarate}] \quad (68)$$

$$\frac{d\text{Glutamate}_{00000}}{dt} = (\alpha\text{Ketoglutarate}_{00000} * J_{\text{exch}} - J_{\text{exch}} * \text{Glutamate}_{00000})/[\text{Glutamate}] \tag{69}$$

$$\frac{d\text{Glutamate}_{00001}}{dt} = (\alpha\text{Ketoglutarate}_{00001} * J_{\text{exch}} - J_{\text{exch}} * \text{Glutamate}_{00001})/[\text{Glutamate}] \tag{70}$$

$$\frac{d\text{Glutamate}_{00010}}{dt} = (\alpha\text{Ketoglutarate}_{00010} * J_{\text{exch}} - J_{\text{exch}} * \text{Glutamate}_{00010})/[\text{Glutamate}] \tag{71}$$

$$\frac{d\text{Glutamate}_{00011}}{dt} = (\alpha\text{Ketoglutarate}_{00011} * J_{\text{exch}} - J_{\text{exch}} * \text{Glutamate}_{00011})/[\text{Glutamate}] \tag{72}$$

$$\frac{d\text{Glutamate}_{00100}}{dt} = (\alpha\text{Ketoglutarate}_{00100} * J_{\text{exch}} - J_{\text{exch}} * \text{Glutamate}_{00100})/[\text{Glutamate}] \tag{73}$$

$$\frac{d\text{Glutamate}_{00101}}{dt} = (\alpha\text{Ketoglutarate}_{00101} * J_{\text{exch}} - J_{\text{exch}} * \text{Glutamate}_{00101})/[\text{Glutamate}] \tag{74}$$

$$\frac{d\text{Glutamate}_{00110}}{dt} = (\alpha\text{Ketoglutarate}_{00110} * J_{\text{exch}} - J_{\text{exch}} * \text{Glutamate}_{00110})/[\text{Glutamate}] \tag{75}$$

$$\frac{d\text{Glutamate}_{00111}}{dt} = (\alpha\text{Ketoglutarate}_{00111} * J_{\text{exch}} - J_{\text{exch}} * \text{Glutamate}_{00111})/[\text{Glutamate}] \tag{76}$$

$$\frac{d\text{Glutamate}_{01000}}{dt} = (\alpha\text{Ketoglutarate}_{01000} * J_{\text{exch}} - J_{\text{exch}} * \text{Glutamate}_{01000})/[\text{Glutamate}] \tag{77}$$

$$\frac{d\text{Glutamate}_{01001}}{dt} = (\alpha\text{Ketoglutarate}_{01001} * J_{\text{exch}} - J_{\text{exch}} * \text{Glutamate}_{01001})/[\text{Glutamate}] \tag{78}$$

$$\frac{d\text{Glutamate}_{01010}}{dt} = (\alpha\text{Ketoglutarate}_{01010} * J_{\text{exch}} - J_{\text{exch}} * \text{Glutamate}_{01010})/[\text{Glutamate}] \tag{79}$$

$$\frac{d\text{Glutamate}_{01011}}{dt} = (\alpha\text{Ketoglutarate}_{01011} * J_{\text{exch}} - J_{\text{exch}} * \text{Glutamate}_{01011})/[\text{Glutamate}] \tag{80}$$

$$\frac{d\text{Glutamate}_{01100}}{dt} = (\alpha\text{Ketoglutarate}_{01100} * J_{\text{exch}} - J_{\text{exch}} * \text{Glutamate}_{01100})/[\text{Glutamate}] \tag{81}$$

$$\frac{d\text{Glutamate}_{01101}}{dt} = (\alpha\text{Ketoglutarate}_{01101} * J_{\text{exch}} - J_{\text{exch}} * \text{Glutamate}_{01101})/[\text{Glutamate}] \tag{82}$$

$$\frac{d\text{Glutamate}_{01110}}{dt} = (\alpha\text{Ketoglutarate}_{01110} * J_{\text{exch}} - J_{\text{exch}} * \text{Glutamate}_{01110})/[\text{Glutamate}] \tag{83}$$

$$\frac{d\text{Glutamate}_{01111}}{dt} = (\alpha\text{Ketoglutarate}_{01111} * J_{\text{exch}} - J_{\text{exch}} * \text{Glutamate}_{01111})/[\text{Glutamate}] \tag{84}$$

$$\frac{d\text{Glutamate}_{10000}}{dt} = (\alpha\text{Ketoglutarate}_{10000} * J_{\text{exch}} - J_{\text{exch}} * \text{Glutamate}_{10000})/[\text{Glutamate}] \tag{85}$$

$$\frac{d\text{Glutamate}_{10001}}{dt} = (\alpha\text{Ketoglutarate}_{10001} * J_{\text{exch}} - J_{\text{exch}} * \text{Glutamate}_{10001})/[\text{Glutamate}] \tag{86}$$

$$\frac{d\text{Glutamate}_{10010}}{dt} = (\alpha\text{Ketoglutarate}_{10010} * J_{\text{exch}} - J_{\text{exch}} * \text{Glutamate}_{10010})/[\text{Glutamate}] \tag{87}$$

$$\frac{d\text{Glutamate}_{10011}}{dt} = (\alpha\text{Ketoglutarate}_{10011} * J_{\text{exch}} - J_{\text{exch}} * \text{Glutamate}_{10011})/[\text{Glutamate}] \tag{88}$$

$$\frac{d\text{Glutamate}_{10100}}{dt} = (\alpha\text{Ketoglutarate}_{10100} * J_{\text{exch}} - J_{\text{exch}} * \text{Glutamate}_{10100})/[\text{Glutamate}] \tag{89}$$

$$\frac{d\text{Glutamate}_{10101}}{dt} = (\alpha\text{Ketoglutarate}_{10101} * J_{\text{exch}} - J_{\text{exch}} * \text{Glutamate}_{10101})/[\text{Glutamate}] \tag{90}$$

$$\frac{d\text{Glutamate}_{10110}}{dt} = (\alpha\text{Ketoglutarate}_{10110} * J_{\text{exch}} - J_{\text{exch}} * \text{Glutamate}_{10110})/[\text{Glutamate}] \tag{91}$$

$$\frac{d\text{Glutamate}_{10111}}{dt} = (\alpha\text{Ketoglutarate}_{10111} * J_{\text{exch}} - J_{\text{exch}} * \text{Glutamate}_{10111})/[\text{Glutamate}] \tag{92}$$

$$\frac{d\text{Glutamate}_{11000}}{dt} = (\alpha\text{Ketoglutarate}_{11000} * J_{\text{exch}} - J_{\text{exch}} * \text{Glutamate}_{11000})/[\text{Glutamate}] \tag{93}$$

$$\frac{d\text{Glutamate}_{11001}}{dt} = (\alpha\text{Ketoglutarate}_{11001} * J_{\text{exch}} - J_{\text{exch}} * \text{Glutamate}_{11001})/[\text{Glutamate}] \tag{94}$$

$$\frac{d\text{Glutamate}_{11010}}{dt} = (\alpha\text{Ketoglutarate}_{11010} * J_{\text{exch}} - J_{\text{exch}} * \text{Glutamate}_{11010})/[\text{Glutamate}] \tag{95}$$

$$\frac{d\text{Glutamate}_{11011}}{dt} = (\alpha\text{Ketoglutarate}_{11011} * J_{\text{exch}} - J_{\text{exch}} * \text{Glutamate}_{11011})/[\text{Glutamate}] \tag{96}$$

$$\frac{d\text{Glutamate}_{11100}}{dt} = (\alpha\text{Ketoglutarate}_{11100} * J_{\text{exch}} - J_{\text{exch}} * \text{Glutamate}_{11100})/[\text{Glutamate}] \quad (97)$$

$$\frac{d\text{Glutamate}_{11101}}{dt} = (\alpha\text{Ketoglutarate}_{11101} * J_{\text{exch}} - J_{\text{exch}} * \text{Glutamate}_{11101})/[\text{Glutamate}] \quad (98)$$

$$\frac{d\text{Glutamate}_{11110}}{dt} = (\alpha\text{Ketoglutarate}_{11110} * J_{\text{exch}} - J_{\text{exch}} * \text{Glutamate}_{11110})/[\text{Glutamate}] \quad (99)$$

$$\frac{d\text{Glutamate}_{11111}}{dt} = (\alpha\text{Ketoglutarate}_{11111} * J_{\text{exch}} - J_{\text{exch}} * \text{Glutamate}_{11111})/[\text{Glutamate}] \quad (100)$$

$$\frac{d\text{Oxaloacetate}_{0000}}{dt} = (\text{Aspartate}_{0000} * J_{\text{exch}} + ((\alpha\text{Ketoglutarate}_{00000} + \alpha\text{Ketoglutarate}_{00001}) * (J_{\text{TCA}}/2) + (\alpha\text{Ketoglutarate}_{00000} + \alpha\text{Ketoglutarate}_{00001}) * (J_{\text{TCA}}/2) + \text{AnapleroticPool}_{0000} * P_{\text{anap}} * J_{\text{TCA}})/(J_{\text{TCA}} + P_{\text{anap}} * J_{\text{TCA}}) * J_{\text{TCA}} - (J_{\text{exch}} + J_{\text{TCA}}) * \text{Oxaloacetate}_{0000})/[\text{Oxaloacetate}] \quad (101)$$

$$\frac{d\text{Oxaloacetate}_{0001}}{dt} = (\text{Aspartate}_{0001} * J_{\text{exch}} + ((\alpha\text{Ketoglutarate}_{00010} + \alpha\text{Ketoglutarate}_{00011}) * (J_{\text{TCA}}/2) + (\alpha\text{Ketoglutarate}_{10000} + \alpha\text{Ketoglutarate}_{10001}) * (J_{\text{TCA}}/2) + \text{AnapleroticPool}_{0001} * P_{\text{anap}} * J_{\text{TCA}})/(J_{\text{TCA}} + P_{\text{anap}} * J_{\text{TCA}}) * J_{\text{TCA}} - (J_{\text{exch}} + J_{\text{TCA}}) * \text{Oxaloacetate}_{0001})/[\text{Oxaloacetate}] \quad (102)$$

$$\frac{d\text{Oxaloacetate}_{0010}}{dt} = (\text{Aspartate}_{0010} * J_{\text{exch}} + ((\alpha\text{Ketoglutarate}_{00100} + \alpha\text{Ketoglutarate}_{00101}) * (J_{\text{TCA}}/2) + (\alpha\text{Ketoglutarate}_{01000} + \alpha\text{Ketoglutarate}_{01001}) * (J_{\text{TCA}}/2) + \text{AnapleroticPool}_{0010} * P_{\text{anap}} * J_{\text{TCA}})/(J_{\text{TCA}} + P_{\text{anap}} * J_{\text{TCA}}) * J_{\text{TCA}} - (J_{\text{exch}} + J_{\text{TCA}}) * \text{Oxaloacetate}_{0010})/[\text{Oxaloacetate}] \quad (103)$$

$$\frac{d\text{Oxaloacetate}_{0011}}{dt} = (\text{Aspartate}_{0011} * J_{\text{exch}} + ((\alpha\text{Ketoglutarate}_{00110} + \alpha\text{Ketoglutarate}_{00111}) * (J_{\text{TCA}}/2) + (\alpha\text{Ketoglutarate}_{11000} + \alpha\text{Ketoglutarate}_{11001}) * (J_{\text{TCA}}/2) + \text{AnapleroticPool}_{0011} * P_{\text{anap}} * J_{\text{TCA}})/(J_{\text{TCA}} + P_{\text{anap}} * J_{\text{TCA}}) * J_{\text{TCA}} - (J_{\text{exch}} + J_{\text{TCA}}) * \text{Oxaloacetate}_{0011})/[\text{Oxaloacetate}] \quad (104)$$

$$\frac{d\text{Oxaloacetate}_{0100}}{dt} = (\text{Aspartate}_{0100} * J_{\text{exch}} + ((\alpha\text{Ketoglutarate}_{01000} + \alpha\text{Ketoglutarate}_{01001}) * (J_{\text{TCA}}/2) + (\alpha\text{Ketoglutarate}_{00100} + \alpha\text{Ketoglutarate}_{00101}) * (J_{\text{TCA}}/2) + \text{AnapleroticPool}_{0100} * P_{\text{anap}} * J_{\text{TCA}})/(J_{\text{TCA}} + P_{\text{anap}} * J_{\text{TCA}}) * J_{\text{TCA}} - (J_{\text{exch}} + J_{\text{TCA}}) * \text{Oxaloacetate}_{0100})/[\text{Oxaloacetate}] \quad (105)$$

$$\frac{d\text{Oxaloacetate}_{0101}}{dt} = (\text{Aspartate}_{0101} * J_{\text{exch}} + ((\alpha\text{Ketoglutarate}_{01010} + \alpha\text{Ketoglutarate}_{01011}) * (J_{\text{TCA}}/2) + (\alpha\text{Ketoglutarate}_{10100} + \alpha\text{Ketoglutarate}_{10101}) * (J_{\text{TCA}}/2) + \text{AnapleroticPool}_{0101} * P_{\text{anap}} * J_{\text{TCA}})/(J_{\text{TCA}} + P_{\text{anap}} * J_{\text{TCA}}) * J_{\text{TCA}} - (J_{\text{exch}} + J_{\text{TCA}}) * \text{Oxaloacetate}_{0101})/[\text{Oxaloacetate}] \quad (106)$$

$$\frac{d\text{Oxaloacetate}_{0110}}{dt} = (\text{Aspartate}_{0110} * J_{\text{exch}} + ((\alpha\text{Ketoglutarate}_{01100} + \alpha\text{Ketoglutarate}_{01101}) * (J_{\text{TCA}}/2) + (\alpha\text{Ketoglutarate}_{01100} + \alpha\text{Ketoglutarate}_{01101}) * (J_{\text{TCA}}/2) + \text{AnapleroticPool}_{0110} * P_{\text{anap}} * J_{\text{TCA}})/(J_{\text{TCA}} + P_{\text{anap}} * J_{\text{TCA}}) * J_{\text{TCA}} - (J_{\text{exch}} + J_{\text{TCA}}) * \text{Oxaloacetate}_{0110})/[\text{Oxaloacetate}] \quad (107)$$

$$\frac{d\text{Oxaloacetate}_{0111}}{dt} = (\text{Aspartate}_{0111} * J_{\text{exch}} + ((\alpha\text{Ketoglutarate}_{01110} + \alpha\text{Ketoglutarate}_{01111}) * (J_{\text{TCA}}/2) + (\alpha\text{Ketoglutarate}_{11100} + \alpha\text{Ketoglutarate}_{11101}) * (J_{\text{TCA}}/2) + \text{AnapleroticPool}_{0111} * P_{\text{anap}} * J_{\text{TCA}})/(J_{\text{TCA}} + P_{\text{anap}} * J_{\text{TCA}}) * J_{\text{TCA}} - (J_{\text{exch}} + J_{\text{TCA}}) * \text{Oxaloacetate}_{0111})/[\text{Oxaloacetate}] \quad (108)$$

$$\frac{d\text{Oxaloacetate}_{1000}}{dt} = (\text{Aspartate}_{1000} * J_{\text{exch}} + ((\alpha\text{Ketoglutarate}_{10000} + \alpha\text{Ketoglutarate}_{10001}) * (J_{\text{TCA}}/2) + (\alpha\text{Ketoglutarate}_{00010} + \alpha\text{Ketoglutarate}_{00011}) * (J_{\text{TCA}}/2) + \text{AnapleroticPool}_{1000} * P_{\text{anap}} * J_{\text{TCA}})/(J_{\text{TCA}} + P_{\text{anap}} * J_{\text{TCA}}) * J_{\text{TCA}} - (J_{\text{exch}} + J_{\text{TCA}}) * \text{Oxaloacetate}_{1000})/[\text{Oxaloacetate}] \quad (109)$$

$$\frac{d\text{Oxaloacetate}_{1001}}{dt} = (\text{Aspartate}_{1001} * J_{\text{exch}} + ((\alpha\text{Ketoglutarate}_{10010} + \alpha\text{Ketoglutarate}_{10011}) * (J_{\text{TCA}}/2) + (\alpha\text{Ketoglutarate}_{10010} + \alpha\text{Ketoglutarate}_{10011}) * (J_{\text{TCA}}/2) + \text{AnapleroticPool}_{1001} * P_{\text{anap}} * J_{\text{TCA}})/(J_{\text{TCA}} + P_{\text{anap}} * J_{\text{TCA}}) * J_{\text{TCA}} - (J_{\text{exch}} + J_{\text{TCA}}) * \text{Oxaloacetate}_{1001})/[\text{Oxaloacetate}] \quad (110)$$

$$\frac{d\text{Oxaloacetate}_{1010}}{dt} = (\text{Aspartate}_{1010} * J_{\text{exch}} + ((\alpha\text{Ketoglutarate}_{10100} + \alpha\text{Ketoglutarate}_{10101}) * (J_{\text{TCA}}/2) + (\alpha\text{Ketoglutarate}_{01010} + \alpha\text{Ketoglutarate}_{01011}) * (J_{\text{TCA}}/2) + \text{AnapleroticPool}_{1010} * P_{\text{anap}} * J_{\text{TCA}})/(J_{\text{TCA}} + P_{\text{anap}} * J_{\text{TCA}}) * J_{\text{TCA}} - (J_{\text{exch}} + J_{\text{TCA}}) * \text{Oxaloacetate}_{1010})/[\text{Oxaloacetate}] \quad (111)$$

$$\frac{d\text{Oxaloacetate}_{1011}}{dt} = (\text{Aspartate}_{1011} * J_{\text{exch}} + ((\alpha\text{Ketoglutarate}_{10110} + \alpha\text{Ketoglutarate}_{10111}) * (J_{\text{TCA}}/2) + (\alpha\text{Ketoglutarate}_{11010} + \alpha\text{Ketoglutarate}_{11011}) * (J_{\text{TCA}}/2) + \text{AnapleroticPool}_{1011} * P_{\text{anap}} * J_{\text{TCA}})/(J_{\text{TCA}} + P_{\text{anap}} * J_{\text{TCA}}) * J_{\text{TCA}} - (J_{\text{exch}} + J_{\text{TCA}}) * \text{Oxaloacetate}_{1011})/[\text{Oxaloacetate}] \quad (112)$$

$$\frac{d\text{Oxaloacetate}_{1100}}{dt} = (\text{Aspartate}_{1100} * J_{\text{exch}} + ((\alpha\text{Ketoglutarate}_{11000} + \alpha\text{Ketoglutarate}_{11001}) * (J_{\text{TCA}}/2) + (\alpha\text{Ketoglutarate}_{00110} + \alpha\text{Ketoglutarate}_{00111}) * (J_{\text{TCA}}/2) + \text{AnapleroticPool}_{1100} * P_{\text{anap}} * J_{\text{TCA}})/(J_{\text{TCA}} + P_{\text{anap}} * J_{\text{TCA}}) * J_{\text{TCA}} - (J_{\text{exch}} + J_{\text{TCA}}) * \text{Oxaloacetate}_{1100})/[\text{Oxaloacetate}] \tag{113}$$

$$\frac{d\text{Oxaloacetate}_{1101}}{dt} = (\text{Aspartate}_{1101} * J_{\text{exch}} + ((\alpha\text{Ketoglutarate}_{11010} + \alpha\text{Ketoglutarate}_{11011}) * (J_{\text{TCA}}/2) + (\alpha\text{Ketoglutarate}_{10110} + \alpha\text{Ketoglutarate}_{10111}) * (J_{\text{TCA}}/2) + \text{AnapleroticPool}_{1101} * P_{\text{anap}} * J_{\text{TCA}})/(J_{\text{TCA}} + P_{\text{anap}} * J_{\text{TCA}}) * J_{\text{TCA}} - (J_{\text{exch}} + J_{\text{TCA}}) * \text{Oxaloacetate}_{1101})/[\text{Oxaloacetate}] \tag{114}$$

$$\frac{d\text{Oxaloacetate}_{1110}}{dt} = (\text{Aspartate}_{1110} * J_{\text{exch}} + ((\alpha\text{Ketoglutarate}_{11100} + \alpha\text{Ketoglutarate}_{11101}) * (J_{\text{TCA}}/2) + (\alpha\text{Ketoglutarate}_{01110} + \alpha\text{Ketoglutarate}_{01111}) * (J_{\text{TCA}}/2) + \text{AnapleroticPool}_{1110} * P_{\text{anap}} * J_{\text{TCA}})/(J_{\text{TCA}} + P_{\text{anap}} * J_{\text{TCA}}) * J_{\text{TCA}} - (J_{\text{exch}} + J_{\text{TCA}}) * \text{Oxaloacetate}_{1110})/[\text{Oxaloacetate}] \tag{115}$$

$$\frac{d\text{Oxaloacetate}_{1111}}{dt} = (\text{Aspartate}_{1111} * J_{\text{exch}} + ((\alpha\text{Ketoglutarate}_{11110} + \alpha\text{Ketoglutarate}_{11111}) * (J_{\text{TCA}}/2) + (\alpha\text{Ketoglutarate}_{11110} + \alpha\text{Ketoglutarate}_{11111}) * (J_{\text{TCA}}/2) + \text{AnapleroticPool}_{1111} * P_{\text{anap}} * J_{\text{TCA}})/(J_{\text{TCA}} + P_{\text{anap}} * J_{\text{TCA}}) * J_{\text{TCA}} - (J_{\text{exch}} + J_{\text{TCA}}) * \text{Oxaloacetate}_{1111})/[\text{Oxaloacetate}] \tag{116}$$

$$\frac{d\text{Aspartate}_{0000}}{dt} = (\text{Oxaloacetate}_{0000} * J_{\text{exch}} - J_{\text{exch}} * \text{Aspartate}_{0000})/[\text{Aspartate}] \tag{117}$$

$$\frac{d\text{Aspartate}_{0001}}{dt} = (\text{Oxaloacetate}_{0001} * J_{\text{exch}} - J_{\text{exch}} * \text{Aspartate}_{0001})/[\text{Aspartate}] \tag{118}$$

$$\frac{d\text{Aspartate}_{0010}}{dt} = (\text{Oxaloacetate}_{0010} * J_{\text{exch}} - J_{\text{exch}} * \text{Aspartate}_{0010})/[\text{Aspartate}] \tag{119}$$

$$\frac{d\text{Aspartate}_{0011}}{dt} = (\text{Oxaloacetate}_{0011} * J_{\text{exch}} - J_{\text{exch}} * \text{Aspartate}_{0011})/[\text{Aspartate}] \tag{120}$$

$$\frac{d\text{Aspartate}_{0100}}{dt} = (\text{Oxaloacetate}_{0100} * J_{\text{exch}} - J_{\text{exch}} * \text{Aspartate}_{0100})/[\text{Aspartate}] \tag{121}$$

$$\frac{d\text{Aspartate}_{0101}}{dt} = (\text{Oxaloacetate}_{0101} * J_{\text{exch}} - J_{\text{exch}} * \text{Aspartate}_{0101})/[\text{Aspartate}] \tag{122}$$

$$\frac{d\text{Aspartate}_{0110}}{dt} = (\text{Oxaloacetate}_{0110} * J_{\text{exch}} - J_{\text{exch}} * \text{Aspartate}_{0110})/[\text{Aspartate}] \tag{123}$$

$$\frac{d\text{Aspartate}_{0111}}{dt} = (\text{Oxaloacetate}_{0111} * J_{\text{exch}} - J_{\text{exch}} * \text{Aspartate}_{0111})/[\text{Aspartate}] \tag{124}$$

$$\frac{d\text{Aspartate}_{1000}}{dt} = (\text{Oxaloacetate}_{1000} * J_{\text{exch}} - J_{\text{exch}} * \text{Aspartate}_{1000})/[\text{Aspartate}] \tag{125}$$

$$\frac{d\text{Aspartate}_{1001}}{dt} = (\text{Oxaloacetate}_{1001} * J_{\text{exch}} - J_{\text{exch}} * \text{Aspartate}_{1001})/[\text{Aspartate}] \tag{126}$$

$$\frac{d\text{Aspartate}_{1010}}{dt} = (\text{Oxaloacetate}_{1010} * J_{\text{exch}} - J_{\text{exch}} * \text{Aspartate}_{1010})/[\text{Aspartate}] \tag{127}$$

$$\frac{d\text{Aspartate}_{1011}}{dt} = (\text{Oxaloacetate}_{1011} * J_{\text{exch}} - J_{\text{exch}} * \text{Aspartate}_{1011})/[\text{Aspartate}] \tag{128}$$

$$\frac{d\text{Aspartate}_{1100}}{dt} = (\text{Oxaloacetate}_{1100} * J_{\text{exch}} - J_{\text{exch}} * \text{Aspartate}_{1100})/[\text{Aspartate}] \tag{129}$$

$$\frac{d\text{Aspartate}_{1101}}{dt} = (\text{Oxaloacetate}_{1101} * J_{\text{exch}} - J_{\text{exch}} * \text{Aspartate}_{1101})/[\text{Aspartate}] \tag{130}$$

$$\frac{d\text{Aspartate}_{1110}}{dt} = (\text{Oxaloacetate}_{1110} * J_{\text{exch}} - J_{\text{exch}} * \text{Aspartate}_{1110})/[\text{Aspartate}] \tag{131}$$

$$\frac{d\text{Aspartate}_{1111}}{dt} = (\text{Oxaloacetate}_{1111} * J_{\text{exch}} - J_{\text{exch}} * \text{Aspartate}_{1111})/[\text{Aspartate}] \tag{132}$$
