# Supplementary material for: Computational estimation of tricarboxylic acid cycle fluxes using noisy NMR data from cardiac biopsies
Source: BMC Syst Biol. 2013 Aug 21;7:82. doi: 10.1186/1752-0509-7-82 (PMC3765389; doi:10.1186/1752-0509-7-82)
Supplement: Additional file 3 — Estimation of auxiliary model parameters. In this supplemental text, the results of estimating the auxiliary model parameters Panap and Jexch are presented and discussed. [file 1752-0509-7-82-S3.pdf]

Supplemental Text: Estimation of auxiliary model parameters

(**Computational estimation of Tricarboxylic Acid Cycle fluxes using noisy NMR data from cardiac biopsies**)

Hannes Hettling, David J.C. Alders, Jaap Heringa, Thomas W. Binsl, A.B. Johan Groeneveld, Johannes H.G.M. van Beek

For the auxiliary model parameters J_{exch} and P_{anap}, accurate point estimates were not expected (see main text). Nevertheless, the uncertainty of the auxiliary parameters was explicitly taken into account in the ensemble sampling. To give an impression of the values and variation of these auxiliary parameters, Figure S1 shows box plots of the distributions as a result of combining all valid parameter ensembles for all samples per experimental condition. Please note that these "grand ensembles" aggregate the variability in the single NMR measurements, the spatial variability between the different samples and the variation amongst different hearts. The "grand median" of the combined distributions for P_{anap} and J_{exch} and also the best-fit values averaged over all samples in all hearts are given in Figure S1. Here we report the median of the "grand ensemble" rather than the mean, because it is more robust when dealing with skewed distributions.

In the control hearts, anaplerosis has a median of the combined ensemble at 31% of J_{TCA}. This is higher than reported by Binsl et al. [1] (see Discussion in the main text). Relative anaplerosis tends to be increased under ischemic conditions: for mild stenosis, moderate stenosis, and the stenosis + adenosine groups, P_{anap} becomes 39%, 71%, and 63% (grand median) of J_{TCA}, respectively. These findings agree with studies on perfused rat hearts which report a higher relative anaplerotic flux during ischemia [2].

The determination of the parameter J_{exch} using ^{13}C MFA is a challenging task [3,4]. As shown previously, the NMR intensities are insensitive to high J_{exch} values and consequently J_{exch} values in the high range cannot be defined accurately. Figure S1 presents a large number of outliers for the estimates of J_{exch} with our ensemble method. For all stress conditions, J_{exch} is found to be lower compared with the control condition. This is corroborated by a lower exchange flux from α-ketoglutarate to glutamate during dobutamine stress previously reported for dog hearts [5]. The "grand median" for J_{exch} for control and dobutamine group was 12.5 and 7.6 μmol/(min*gdw) which is in agreement with previous estimates by Binsl et al. [1].

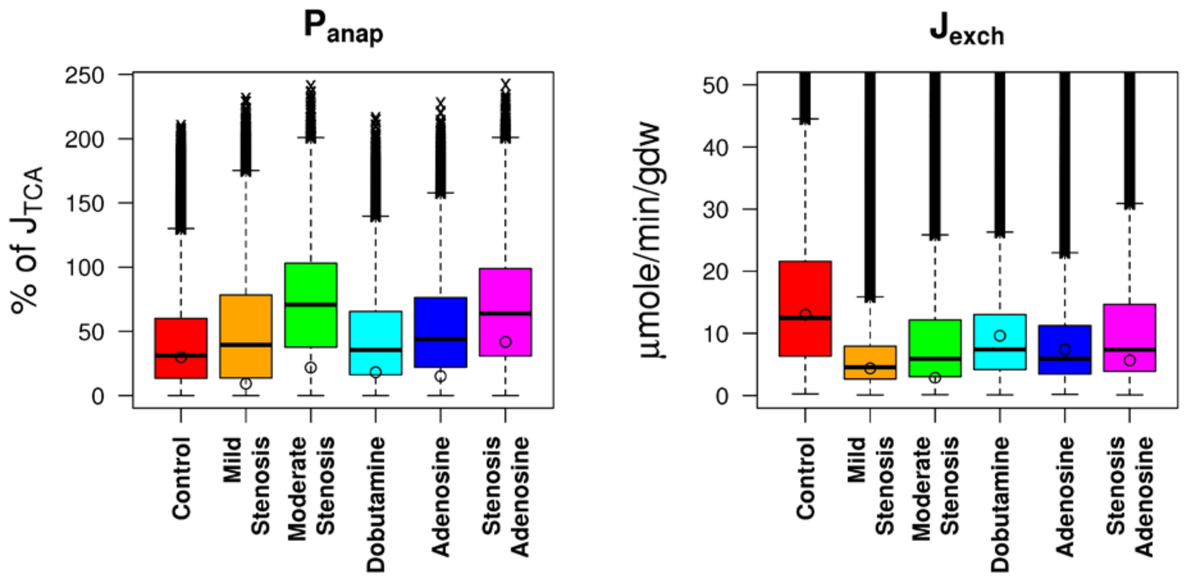

Figure S1: ***Composite ensemble distributions for the auxiliary parameters P_{anap} and J_{exch} representing relative anaplerosis and the exchange flux between TCA cycle intermediates and amino acids, respectively. Colored boxes show the interquartile range of the distributions and their medians. The ends of the whiskers stand for the lowest and highest value still within 1.5 times the interquartile range of the lower and upper quartile, respectively. Best-fit parameter values averaged over all samples and all hearts per group are plotted as open circles. Outliers are plotted with the symbol "x". Note that in the right hand plot outliers above 50 µmole/(min*gdw) have been omitted.***

REFERENCES

1. Binsl TW, Alders DJC, Heringa J, Groeneveld ABJ, van Beek JHGM: **Computational quantification of metabolic fluxes from a single isotope snapshot: application to an animal biopsy.** *Bioinformatics* 2010, **26** (5):653–660.

2. Lloyd SG, Wang P, Zeng H, Chatham JC: **Impact of low-flow ischemia on substrate oxidation and glycolysis in the isolated perfused rat heart.** *Am J Physiol Heart Circ Physiol* 2004, **287**(1):H351–362.

3. Chance EM, Seeholzer SH, Kobayashi K, Williamson JR: **Mathematical analysis of isotope labeling in the citric acid cycle with applications to 13C NMR studies in perfused rat hearts.** *J Biol Chem* 1983, **258** (22):13785–13794.

4. Jeffrey FMH, Reshetov A, Storey CJ, Carvalho RA, Sherry AD, Malloy CR: **Use of a single 13C NMR resonance of glutamate for measuring oxygen consumption in tissue**. *Am J Physiol* 1999, **277**(6 Pt 1):E1111–1121.

5. O'Donnell JM, Kudej RK, LaNoue KF, Vatner SF, Lewandowski ED: **Limited transfer of cytosolic NADH into mitochondria at high cardiac workload.** *Am J Physiol Heart Circ Physiol* 2004, **286**(6):H2237–2242.
